# Bioprinted Patient‐Derived Organoid Arrays Capture Intrinsic and Extrinsic Tumor Features for Advanced Personalized Medicine

**DOI:** 10.1002/advs.202407871

**Published:** 2025-03-28

**Authors:** Jonghyeuk Han, Hye‐Jin Jeong, Jeonghan Choi, Hyeonseo Kim, Taejoon Kwon, Kyungjae Myung, Kyemyung Park, Jung In Park, Samuel Sánchez, Deok‐Beom Jung, Chang Sik Yu, In Ho Song, Jin‐Hyung Shim, Seung‐Jae Myung, Hyun‐Wook Kang, Tae‐Eun Park

**Affiliations:** ^1^ Department of Biomedical Engineering Ulsan National Institute of Science and Technology (UNIST) Ulsan 44919 Republic of Korea; ^2^ Wallace H. Coulter Department of Biomedical Engineering Emory University School of Medicine & Georgia Institute of Technology Atlanta GA 30332 USA; ^3^ Center for Genome Engineering Institute for Basic Science Daejeon 34126 Republic of Korea; ^4^ Center for Genomic Integrity Institute for Basic Science Ulsan 44919 Republic of Korea; ^5^ Graduate School of Health Science and Technology and Department of Biomedical Engineering Ulsan National Institute of Science and Technology Ulsan 44919 Republic of Korea; ^6^ Institute for Bioengineering of Catalonia (IBEC) The Barcelona Institute for Science and Technology (BIST) Barcelona 08028 Spain; ^7^ Catalan Institute for Research and Advanced Studies (ICREA) Barcelona 08010 Spain; ^8^ Digestive Diseases Research Center University of Ulsan College of Medicine Seoul 05505 Republic of Korea; ^9^ Division of Colon and Rectal Surgery Department of Surgery Asan Medical Center University of Ulsan College of Medicine Seoul 05505 Republic of Korea; ^10^ Research Institute T&R Biofab Co. Ltd. Siheung 15111 Republic of Korea; ^11^ Department of Mechanical Engineering Tech University of Korea Siheung 15073 Republic of Korea; ^12^ Department of Gastroenterology Asan Medical Center University of Ulsan College of Medicine Seoul 05505 Republic of Korea; ^13^ EDIS Biotech Seoul 05505 Republic of Korea

**Keywords:** colorectal cancer, embedded bioprinting, inter‐patient variability, patient‐derived tumor organoid, supervised learning, tumor matrix stiffness

## Abstract

Heterogeneity and the absence of a tumor microenvironment (TME) in traditional patient‐derived organoid (PDO) cultures limit their effectiveness for clinical use. Here, Embedded Bioprinting‐enabled Arrayed PDOs (Eba‐PDOs) featuring uniformly arrayed colorectal cancer (CRC) PDOs within a recreated TME is presented. This model faithfully reproduces critical TME attributes, including elevated matrix stiffness (≈7.5 kPa) and hypoxic conditions found in CRC. Transcriptomic and immunofluorescence microscopy analysis reveal that Eba‐PDOs more accurately represent actual tissues compared to traditional PDOs. Furthermore, Eba‐PDO effectively capture the variability of CEACAM5 expression—a critical CRC marker—across different patients, correlating with patient classification and differential responses to 5‐fluorouracil treatment. This method achieves an uniform size and shape within PDOs from the same patient while preserving distinct morphological features among those from different individuals. These features of Eba‐PDO enable the efficient development of a label‐free, morphology‐based predictive model using supervised learning, enhancing its suitability for clinical applications. Thus, this approach to PDO bioprinting is a promising tool for generating personalized tumor models and advancing precision medicine.

## Graphical abstract

An embedded bioprinting enabled‐arrayed patient‐derived organoids (Eba‐PDO) platform that replicates intrinsic and extrinsic tumor characteristics is introduced. Eba‐PDOs more accurately mimic tissue than standard PDOs (Std‐PDOs) due to maturation in the tumor microenvironment. This technology also ensures uniformity in size and shape while preserving distinct features between patients, enabling a label‐free, morphology‐based predictive model, thus facilitating faster, reliable medical guidance.

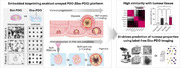

## 1 Introduction

The high failure rates of drug candidates during the clinical approval processes is attributed to significant gaps in preclinical research, particularly in oncology.^[^ ^1^ ^]^ Traditional testing platforms often use overly simplified systems that do not completely capture the complexities of human pathology, particularly cancer. Cancer progression, treatment response, and clinical outcomes are influenced by a mix of intrinsic factors, such as genomic instability, mutations, and epigenetic changes, and extrinsic factors, tumor microenvironment (TME) interactions. This includes communication with immune cells and fibroblasts and the physical properties of the extracellular matrix (ECM), such as stiffness and hypoxia. These dynamics within the TME significantly affect the therapeutic responses,^[^ ^2^ ^]^ underscoring the urgent need for more comprehensive preclinical tumor models that reflect these complex interactions.

Patient‐derived organoids (PDOs) have become a significant tool for reflecting the genetic diversity of cancer cells in vivo,^[^ ^3^ ^]^ with high fidelity in mimicking the morphology and therapeutic responses of actual tumors.^[^ ^4^ ^]^ However, culturing patient‐derived tumor cells in 3D animal‐derived extracellular matrix (ECM) hydrogels, such as Matrigel and Geltrex, often leads to PDO formation with varying sizes and shapes. This variability, stemming from the cells‘ self‐assembling nature within the matrix, challenges the consistency of experimental outcomes. Moreover, although these ECM hydrogels provide the necessary biochemical support for growth, they do not replicate key tumor properties, such as the increased stiffness, potentially causing nonphysiological cell behaviors and unreliable drug response predictions.^[^ ^2^ ^]^

Various strategies have been developed to enhance the influence of external factors on cancer cell 3D cultures. Techniques such as synthetic hydrogels, microfluidic systems, and 3D bioprinting aim to replicate critical aspects of TME.^[^ ^5^ ^]^ Studies on PDOs using these technologies have demonstrated behavioral changes within TME.^[^ ^6^ ^]^ For instance, pancreatic ductal adenocarcinoma (PDAC) PDOs exhibit increased drug resistance when grown in stiff synthetic matrices that mimic the TME, although not completely matching the natural tissue stiffness.^[^ ^6a^ ^]^ Additionally, exposing PDAC PDOs to hypoxic conditions via microfluidic systems alters their drug response.

Although 3D bioprinting has the potential to recreate tumor tissues and precisely control external factors, its full application in PDO research is still being investigated. A key challenge in traditional extrusion‐based PDO bioprinting is maintaining high PDO viability, while achieving reproducible geometrical details and biophysicochemical TME features.^[^ ^7^ ^]^ Traditional PDO culture matrices such as Matrigel or Geltrex, crucial for supporting PDO growth, exhibit suboptimal printability due to their low viscosity. Recent advances with decellularized ECM‐based bioinks have improved printability for extrusion‐based PDO printing, efficiently supported PDO cell growth, and even mimicked the stiffness of tumor tissues.^[^ ^8^ ^]^ Despite these improvements, achieving uniformity in PDOs remains a challenge.^[^ ^8^ ^]^ Additionally, previous studies have successfully produced uniform PDO droplets for bladder and colorectal cancers (CRC)^[^ ^6e^ ^]^ using acoustic droplet bioprinting, but these droplets still require transfer to Matrigel to sustain growth.

Here, we present an approach that utilizes embedded bioprinting technology to create Embedded Bioprinting‐enabled Arrayed PDOs (Eba‐PDOs), which faithfully replicate both intrinsic and extrinsic tumor characteristics while maintaining uniformity. This technology addresses the previous limitations of PDO printing by directly extruding Geltrex‐based PDO‐inks within a supportive alginate hydrogel bath. This enables precise placement of PDO‐inks into organized arrays and supports the formation of uniform 3D tumor structure within an optimal Geltrex environment, confined by the alginate bath. The bath‐inks, engineered to replicate tumor stiffness and low‐oxygen conditions, enable the Eba‐PDOs to more closely mimic the original tumor tissue compared to standard PDO cultures (Std‐PDOs). We also highlighted that the Eba‐PDO platform could create a label‐free morphometric analysis‐based prediction model that enables faster and more personalized medical guidance.

## 2 Results and Discussion

### 2.1 Embedded Bioprinting Producing Uniform Arrayed CRC PDOs

**Figure 1:**
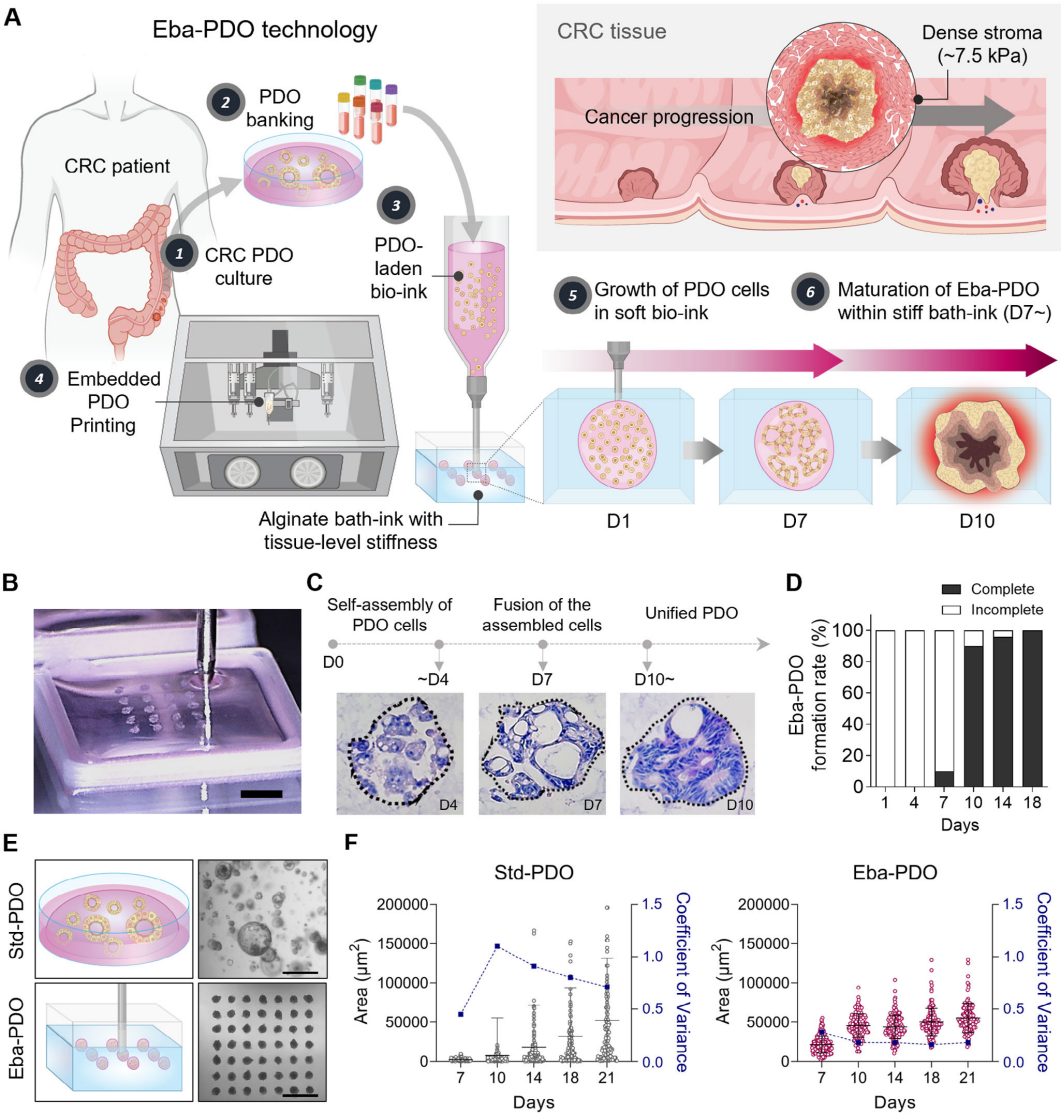
Creating tumor microenvironment (TME)‐inspired embedded bioprinting‐enabled arrayed patient‐derived organoids (Eba‐PDOs). A) A schematic illustrating embedded 3D bioprinting process of PDO‐ink based on a Geltrex™ hydrogel within an alginate bath, designed to mimic the natural colorectal cancer (CRC) tissue surrounded by a rigid matrix, highlighting its importance in cancer progression. B) Photograph showing the dispensation of PDO‐laden bio‐ink into an alginate bath (Scale bar = 1 mm). C) Haematoxylin‐eosin (H&E) staining images displaying Eba‐PDO formation within the alginate bath: self‐assembly of suspended PDO cells on day 4, fusion of assembled PDO cells on day 7, and unified Eba‐PDO formation on day 10 (Scale bar = 50 µm). D) Formation rate of a unified Eba‐PDO over time. E) A comparative illustration (left) and bright‐field microscopy images (right) display the differences between standard PDOs (Std‐PDO) within a Geltrex™ dome and Eba‐PDOs on day 14. Scale bar = 500 µm. F) A comparison of the area and variance of Std‐PDOs and Eba‐PDOs throughout the growth period. Data were obtained using PDOs derived from a single CRC patient (CEA_lo_‐11, CEA_lo_: Low‐CEACAM5). The results are presented as mean ± SEM.

This study presents a novel embedded PDO printing method that replicates the compressive stress experienced by tumor cells within a dense stroma during tumor growth (**Figure** **1**A). This method involves extruding PDO‐ink, which is primarily composed of dissociated PDO cells in Geltrex and a gelatin/hyaluronic acid (HA) mixture, into an alginate bath, designed to provide mechanical stimuli to PDO cells (Figure 1B). The alginate bath mimics the stiffness similar to CRC tissues (≈7.5 kPa) using a 1.5% alginate concentration.

Initially, we adjusted the Geltrex composition in PDO‐ink to enhance growth and morphogenesis of dissociated PDO cells via providing an optimal ECM for cultivation. Geltrex concentrations were assessed at 0%, 25%, 50%, and 75% (v/v), which revealed that a 50% (v/v) Geltrex concentration was optimal for supporting cell growth and forming an integrated PDO structure (Figure S1, Supporting Information). We further refined the dissociated PDO cell density and determined that 50 × 10^6^ cells mL^−1^ was ideal for consistent PDO formation and effective printing. These cells form uniform PDOs (≈200 µm) within seven days when positioned within the alginate boundary, self‐assembling and subsequently fusing into a unified PDO by day 10 (Figure 1C,D; Figure S1, Supporting Information). In PDO‐ink, 32.5 mg mL^−1^ gelatin was included for its shear‐thinning properties, which aided in smooth printing. Its temperature‐sensitive viscosity enabled precise positioning within the alginate bath. The PDO‐inks composed of gelatin, HA, and Geltrex—rich in laminin—created a cancer‐specific biochemical environment for the PDOs by supplementing the ECM components lack in the alginate bath. Laminin, a major component of the cancer basement membrane, was shown to surround and adhere to the PDOs at the alginate boundary, mimicking the tissue structure of CRC^[^ ^9^ ^]^ (Figure S2, Supporting Information). Notably, as laminin levels decreased and collagen I levels rose by day 10, it suggests that the PDOs actively remodel their ECM surroundings during self‐assembly.

Comparatively, traditional extrusion bioprinting methods, which suspend dissociated PDO cells directly into the 1.5% alginate bath‐ink to create uniform droplets, failed to form unified PDOs (Figure S3, Supporting Information), probably because biological motifs providing structural and biochemical signals to cells were absent in the alginate bath‐ink, which could disrupt PDO cell growth in their early phases. These findings emphasize the importance of embedded PDO printing technology for successful early growth and maturation of PDOs.

Further assessments revealed that the embedded bioprinting technique produced uniformly sized PDOs, in contrast to the traditional PDO culture method and micromold systems^[^ ^10^ ^]^ (Figure 1E; Figures S4 and S5, Supporting Information). The Eba‐PDOs maintained a stable area from day 10 to 21, which was an advantage attributed to the alginate confining properties. Eba‐PDO uniformity was superior, demonstrating a lower coefficient of variance compared to Std‐PDOs (0.18 versus 0.71 at day 21), which displayed time‐dependent enlargement, showed variable sizes, and sometimes incomplete formations (Figure 1F). The aspect ratios of Std‐PDO and Eba‐PDO shapes did not exhibit significant differences (Figure S4, Supporting Information). In the micromould system, integrated PDOs were partially formed without uniformity, likely due to significant cell loss in the culture medium (Figure S5, Supporting Information). We assume that cystic PDOs float in the medium because of their low density and remain unstably positioned in the micromould, suggesting that the embedded bioprinting method, which retains PDO cells within alginate confines, is more effective in maintaining cell positioning and stability.

### 2.2 Eba‐PDOs Mimic Cancer‐Specific Morphology and Pathophysiology

**Figure 2:**
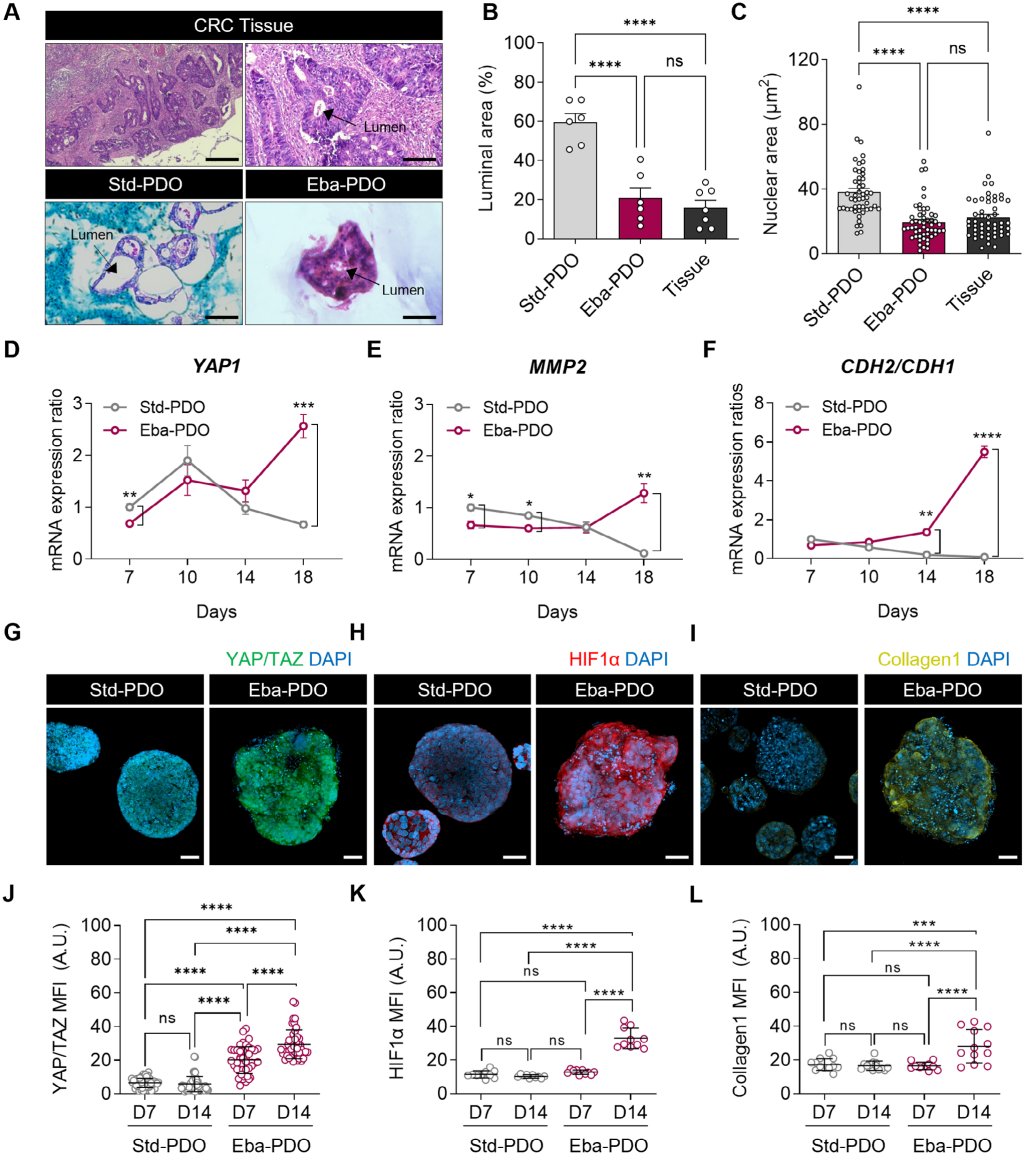
Characterization of cancer hallmarks including morphological features and pathophysiological environments in Eba‐PDOs. A) H&E staining images displaying the morphological features of native CRC tissue, Std‐PDOs, and Eba‐PDOs (Scale bar = 100 µm (CRC Tissue left), 25 µm (CRC Tissue right, Std‐PDO, and Eba‐PDO)). B,C) Analysis of the proportion of luminal area (B) (*n* = 6 for Std‐PDOs, Eba‐PDOs and 7 for Tissues) and nuclear area (C) of native CRC tissue, Std‐PDOs, and Eba‐PDOs (*n* = 47 for Std‐PDOs, 52 for Eba‐PDOs, and 59 for Tissues). D–F) The relative mRNA expression of *YAP1* (D), *MMP2* (E), and *CDH2* and *CDH1* mRNA ratio (*CDH2*/*CDH1*) indicating the epithelial‐mesenchymal transition (F) throughout the PDO culture period. The quantitative real time polymerase chain reaction results are presented as mean ± SEM. For statistical analysis, Student's *t*‐test was performed between each culture day (*n* = 3 for Std‐PDOs and *n* = 6 for Eba‐PDOs). G–I) Immunofluorescence micrographic images representing the cancer pathophysiological environment including YAP/TAZ (indicative of mechanical stress) (G), HIF1α (indicative of hypoxia) (H), and Collagen I (indicative of ECM remodelling) (I). J–L) Mean fluorescent intensity (MFI) of immunofluorescence micropraphic images labeled with YAP/TAZ (J), HIF1α (K), and COL1A1 (L) in Std‐PDOs and Eba‐PDOs on days 7 and 14 (Scale bar = 50 µm). The absence of nonspecific staining from antibody binding was confirmed in Figure S8 (Supporting Information). Results are presented as mean ± SEM. For statistical analysis, a one‐way analysis of variance and Tukey's test were performed (^*^ *p* < 0.05, ^**^ *p* < 0.01, ^***^ *p* < 0.001, ^****^ *p* < 0.0001, ns: non‐significant for all statistical analysis). Data were obtained using PDOs and tissues derived from a single CRC patient (CEA_lo_‐11).

The effect of the Eba‐PDO environment, which replicated tumor‐like external compression using an alginate bath, mirroring the cancer‐specific phenotypes was explored. A dense stromal environment and uncontrolled cell proliferation causes cell jamming in tumor tissues.^[^ ^11^ ^]^ We analyzed haematoxylin‐eosin‐stained sections to observe the morphological distinctions between Std‐PDOs and Eba‐PDOs, compared to those of the original CRC tissue (**Figure** **2**A). Eba‐PDOs had clearly smaller cyst structures, ≈4 times smaller than those of Std‐PDOs (Figure 2B). Nuclear areas were reduced by ≈1.7‐fold, indicating deformation due to confining stress, a common observation in patients with CRC^[^ ^12^ ^]^ (Figure 2C).

Additionally, we examined genes associated with tumor progression under mechanical stress and hypoxia (Figure 2D–F). *YAP1* is a key mechanotransducer that senses mechanical stimuli, which showed a fourfold increase in its expression in Eba‐PDOs by day 18 compared with that in Std‐PDOs (Figure 2D). Confocal immunofluorescence microscopy also revealed a significant upregulation of YAP/TAZ protein levels in Eba‐PDOs between days 7 and 14, whereas Std‐PDOs maintained stable levels (Figure 2G,J). This pattern mirrors YAP overexpression observed in ≈85% patients with CRC,^[^ ^13^ ^]^ suggesting that Eba‐PDOs are responsive to mechanical stresses of the embedded bioprinting platform. Further analysis indicated significant changes in Eba‐PDOs in response to matrix stiffness, including an increase in *MMP2* expression on day 18.^[^ ^14^ ^]^ MMP2, an enzyme involved in ECM remodeling, is expressed at higher levels in 99% of CRC patients compared to normal mucosa and is associated with poorer CRC outcomes^[^ ^15^ ^]^ (Figure 2E). Furthermore, the ratio of *CDH2* to *CDH1* expression, indicating the epithelial‐mesenchymal transition phenotype and metastasis^[^ ^16^ ^]^—often stimulated by matrix stiffness^[^ ^17^ ^]^—was consistently increased in Eba‐PDOs (Figure 2F).

We also noted elevated levels of HIF1α protein (Figure 2H,K) and BioTracker 520 Green hypoxia dye (Figure S6, Supporting Information) in Eba‐PDOs. This is likely due to cell jamming within the confined boundary in alginate, leading to a 2.49‐fold increase in cell density compared to Std‐PDOs (Figure S7, Supporting Information). This indicates that our model could effectively emulate several aspects of the native tumor environment, including modulated cancer cell metabolism and therapeutic resistance, which are typically observed in hypoxic CRC tissues.^[^ ^18^ ^]^

We also investigated type I collagen expression in Eba‐PDOs. Previous studies have noted that tumor cells synthesize modest quantities of collagen, creating a specialized ECM environment within TME.^[^ ^19^ ^]^ By day 14, Eba‐PDOs showed a pronounced accumulation of collagen I, unlike in Std‐PDOs, which showed no detectable expression (Figure 2I,L). These findings indicate the efficacy of the embedded 3D bioprinting method, with its CRC‐analogous stiffness (≈7.5 kPa) and simulated hypoxic conditions, in recreating specific morphological and pathophysiological cancer features.

### 2.3 Transcriptomic Analysis of Eba‐PDOs

**Figure 3:**
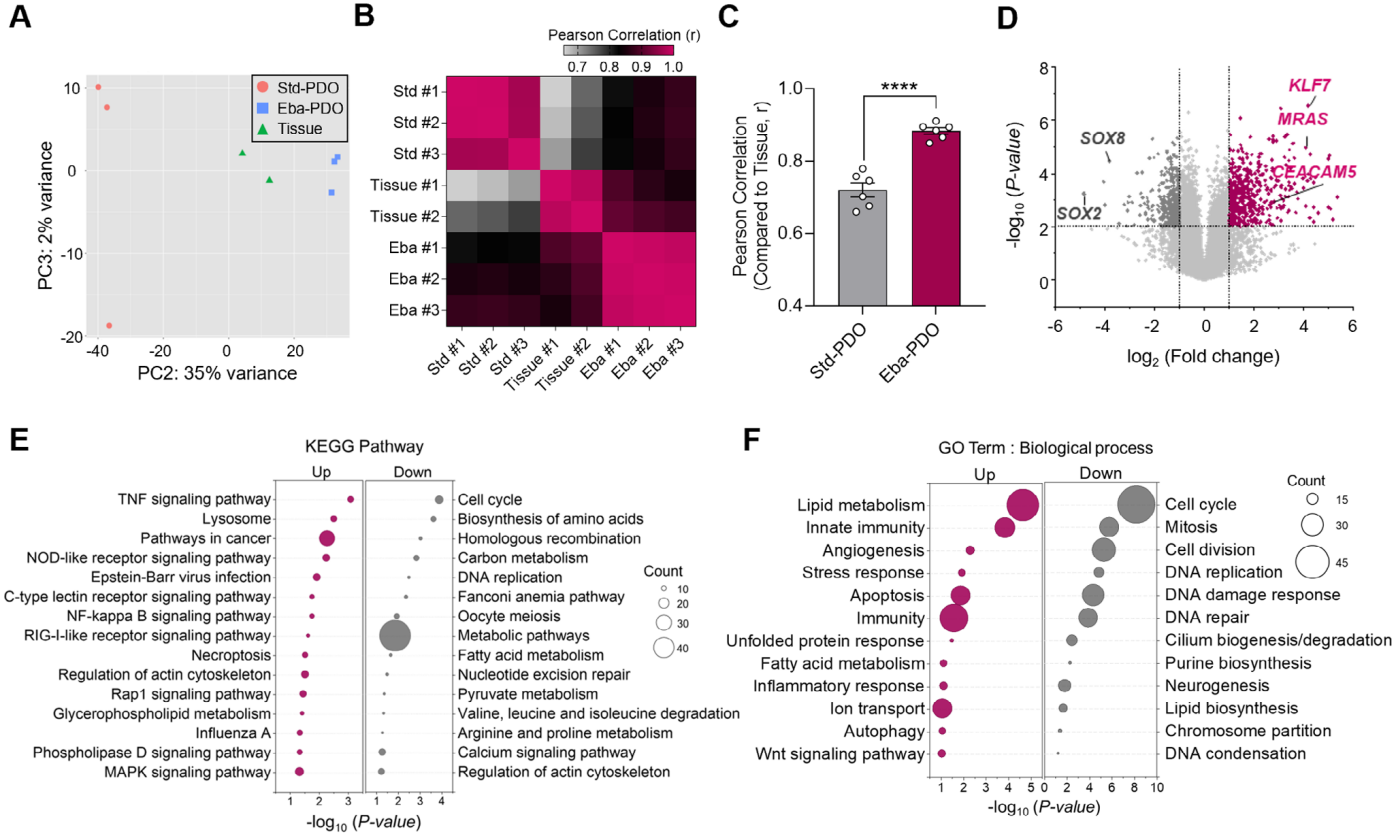
Transcriptomic comparison of Eba‐PDOs compared to Std‐PDOs and the native CRC tissue (Tissue). A) Principal component analysis (PCA) with normalized counts from RNA‐seq data of all samples. B) Pearson”s correlation analysis with most frequently mutated 1004 genes in CRC from National Cancer Institute (https://portal.gdc.cancer.gov/). C) Comparison of Pearson correlation coefficient (r) values between Std‐PDO and Eba‐PDO platforms compared to the CRC tissue. The results are presented as mean ± SEM. For statistical analysis, Student”s *t*‐test was performed between each group (*n* = 6 for Std‐PDOs and Eba‐PDOs, ^****^ *p* < 0.0001). Results are D) Volcano plot showing fold changes for genes differentially expressed between Std‐ and Eba‐PDOs. Genes upregulated and downregulated in Eba‐PDOs compared to Std‐PDOs are highlighted in magenta, and dark grey, respectively. *p* < 0.01. E) Kyoto Encyclopaedia of Genes and Genomes (KEGG) functional classification, and F) Gene Ontology (GO) terms of biological process showing genes upregulated and downregulated in Eba‐PDOs compared to Std‐PDOs. The size of each dot represents the number of differential genes in the enrichment pathway. D–F) Comparison of Differentially‐Expressed Genes (DEGs) between Eba‐PDOs and Std‐PDOs focuses on genes that display a similar expression pattern between the Eba‐PDOs and the original tissue samples. Data were obtained using PDOs and tissues derived from a single CRC patient (CEA_hi_‐02).

To further understand transcriptional variations influenced by different external conditions, we analyzed the transcriptomes of Std‐PDOs, Eba‐PDOs, and CRC tissues from the same patient. Principal component analysis (PCA) clearly distinguished the three groups, with the second principal component (PC2) driving significant transcriptional differences. The PCA result showed that Eba‐PDOs exhibited greater transcriptional similarity to native CRC tissues compared to Std‐PDOs (**Figure** **3**A). We also performed Pearson correlation analyses on the transcriptomic data, focusing on 1004 genes that were most frequently mutated in CRC based on NCI Genomic Data Commons (https://portal.gdc.cancer.gov/) data (Table S1, Supporting Information).^[^ ^20^ ^]^ This analysis confirmed that Eba‐PDOs matched the CRC tissue significantly more closely than Std‐PDOs (Figure 3B,C).

To elucidate the molecular mechanisms that explain the more accurate mirroring of native tissues by Eba‐PDOs, we analyzed differentially expressed genes (DEGs) between PDO platforms, specifically targeting genes with similar expression profiles in Eba‐PDOs and original CRC tissues (Table S2, Supporting Information). This analysis identified 1470 DEGs, with 769 upregulated and 701 downregulated genes in Eba‐PDOs compared to Std‐PDOs (Figure 3D). Notably, the upregulated transcription factor *KLF7*, associated with a poor prognosis in CRC,^[^ ^21^ ^]^ is involved in hypoxia‐mediated gene regulation.^[^ ^22^ ^]^ Additionally, the increase in *MRAS*, a target of the mechanosensor YAP,^[^ ^23^ ^]^ indicates that the Eba‐PDOs respond to the stiff alginate environment. Another significant finding is the amplification of *CEACAM5*, a crucial CRC biomarker,^[^ ^24^ ^]^ known to be influenced by hypoxia and matrix stiffness.^[^ ^25^ ^]^

Conversely, the most downregulated genes were *SOX2* and *SOX8*, suggesting a reduction in poorly differentiated cells^[^ ^26^ ^]^ in Eba‐PDOs (Figure 3D). This change indicates a shift away from the typical overabundance of cancer stem cells promoted by traditional PDO culture conditions, a trend also noted in PDOs cultured in micromolds in previous studies.^[^ ^10b^ ^]^ These findings support that Eba‐PDOs more accurately reflect tumor characteristics.

Pathway enrichment analysis using the Kyoto Encyclopaedia of Genes and Genomes (KEGG) showed that DEGs were primarily involved in inflammatory pathways crucial for CRC pathogenesis and metastasis, such as tumor necrosis factor (TNF), nod‐like receptor, NF‐kappa B, and C‐type lectin receptor‐mediated signalling (Figure 3E). This implies that Eba‐PDOs may provide a more physiologically relevant environment for studying immune cell infiltration, which is critical in CRC prognosis.^[^ ^27^ ^]^ These DEGs were prevalent in cancer pathways, highlighting the impact of TME simulated in Eba‐PDOs. Cell cycle‐associated pathways were also suppressed, suggesting that Std‐PDOs may overemphasize proliferative features that were uncharacteristic of CRC^[^ ^28^ ^]^ (Figure 3E). The most enriched biological process gene ontology (GO) terms were related to lipid metabolism (Figure 3F), which was a key aspect of CRC pathology that enabled survival under conditions such as hypoxia and nutrient scarcity.^[^ ^29^ ^]^

A detailed analysis of DEGs between Std‐ and Eba‐PDOs, aimed at identifying limitations of Eba‐PDOs via comparing genes that were similarly expressed in Std‐PDOs and original CRC tissues, revealed no significant alterations in genes fundamentally linked to CRC (Table S1, Supporting Information). These results underscore the high fidelity of the Eba‐PDO platform in replicating the transcriptional landscape of native CRC tissues, highlighting its potential as an accurate cancer research model. Comparative analysis of DEGs between the tissues and both PDO platforms revealed a deficiency in immunity‐related biological processes in PDO platforms because immune cells were absent. This suggests the importance of integrating immune cells into the platform for future enhancements to improve its fidelity (Figure S9, Supporting Information).

### 2.4 Matrix Stiffness on CEACAM5 Levels and Patterns in Eba‐PDOs

**Figure 4:**
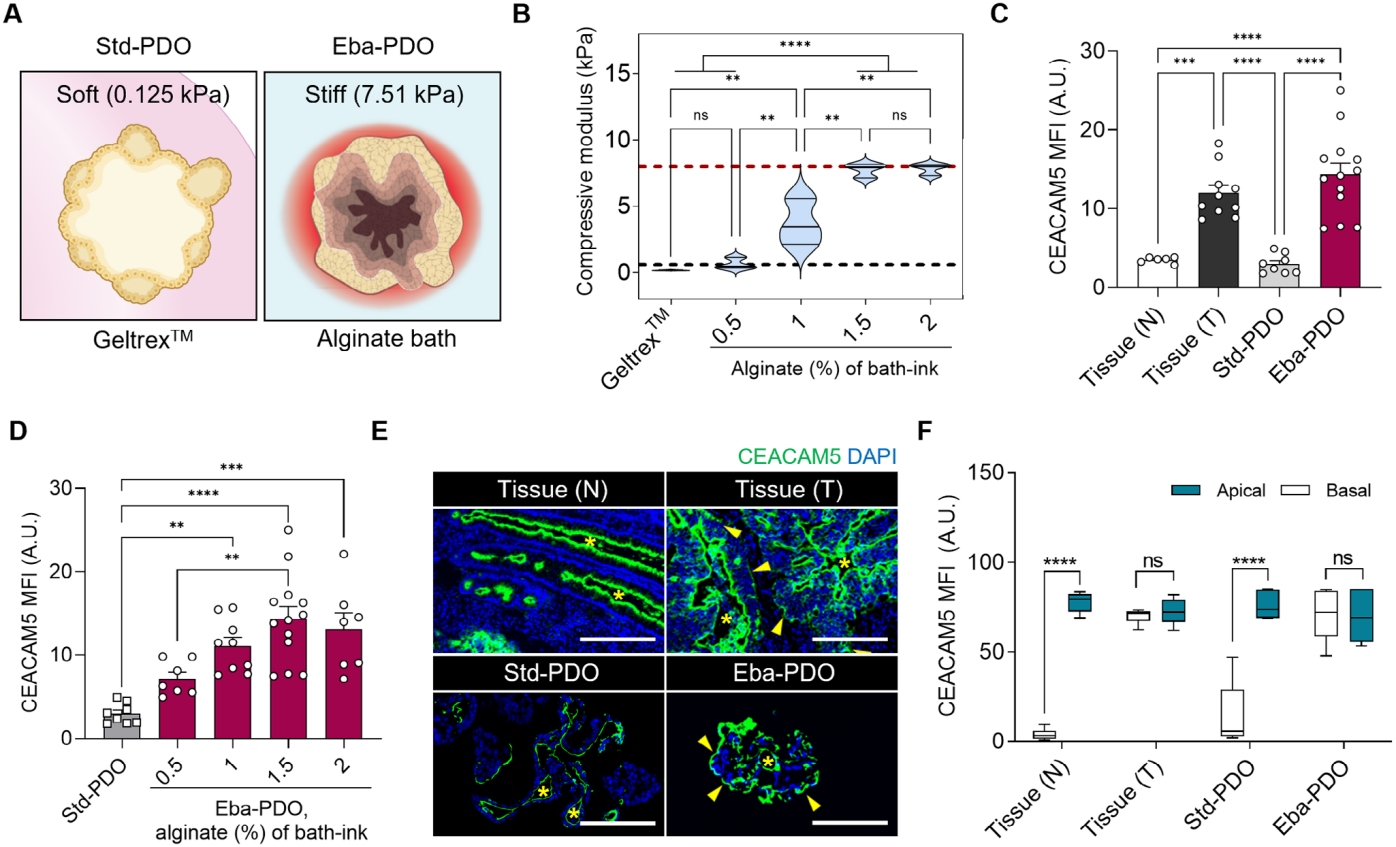
Patterns of CEACAM5 expression in Eba‐PDOs influenced by matrix stiffness. A) A schematic representation of Std‐ and Eba‐PDOs in matrices of differing stiffness. B) The compressive modulus of alginate bath‐ink across different alginate concentrations; the red line shows the median compressive modulus of human CRC tissues and the green line indicates that of normal colorectal tissue (*n* = 3; independent experiments). C) Quantification of mean fluorescence intensity (MFI) within specified regions of interest (ROIs) in confocal immunofluorescent images of sectioned PDOs and normal (N) and tumor tissue (T) sections from the same patients marked with CEACAM5 (*n* = 6 for Tissue (N), 10 for Tissue (T), 8 for Std‐PDOs, and 13 for Eba‐PDOs, A.U.: Arbitrary Unit). Eba‐PDOs were prepared using 1.5% alginate bath‐ink. D) Quantification of MFI within ROI in confocal immunofluorescent images of sectioned Std‐PDOs and Eba‐PDOs marked with CEACAM5, across different alginate bath conditions (*n* = 8 for Std‐PDOs, 7 for Eba‐PDOs (0.5%), 9 for Eba‐PDOs (1%), 13 for Eba‐PDOs (1.5%), 7 for Eba‐PDOs (2%). E) Confocal immunofluorescence microscopic analysis of sectioned PDOs and tissue sections from the same patient, labeled with DAPI (blue) and CEACAM5 (green). Yellow arrows highlight CEACAM5 expression on the abluminal side of normal colon and CRC tissues, and PDOs, with their abluminal side defined as the side oriented toward the matrix. Yellow asterisks represent the lumen of tissues or PDOs. Scale bar = 250 µm. F) The MFI of sectioned PDOs and tissues was measured for ROIs designated as luminal and abluminal regions (*n* = 7 for Tissue (N), 6 for Tissue (T), 6 for Std‐PDOs, and 7 for Eba‐PDOs). All data are presented as mean ± SEM. Statistical significance was determined using a one‐way analysis of variance and Tukey's test (^*^ *p* < 0.05; ^**^ *p* < 0.01; ^***^ *p* < 0.001; ^****^ *p* < 0.0001, ns: non‐significant). Data was obtained using PDOs and tissues derived from a single CRC patient (CEA_lo_‐11).

Transcriptomic analysis revealed that external stimuli within the alginate bath generally promoted tumor progression, hence more accurately presenting CRC. Specifically, *CEACAM5* mRNA expression in Eba‐PDOs was significantly elevated compared to that in Std‐PDOs, closely resembling that in the original CRC tissue. Carcinoembryonic antigen (CEACAM5, CEA) is highly expressed in ≈90% CRC cases and plays an important role in cell metastasis and chemosensitivity.^[^ ^30^ ^]^ CEACAM5 levels are closely associated with progressive features of CRC; hence, it is a crucial biomarker.^[^ ^31^ ^]^ We further monitored *CEACAM5* expression in PDOs in different alginate bath conditions to further understand the impact of external stimuli on PDO (**Figure** **4**A).

Initially, we determined that the alginate compressive modulus could be adjusted via varying its concentration between 0.5% and 2%. Compressive moduli of 0.5%, 1%, 1.5%, and 2% alginate were 0.48, 3, 7.5, and 8 kPa, respectively. The alginate concentration was set to 1.5% to replicate the CRC tissue stiffness level (7.51 kPa),^[^ ^32^ ^]^ whereas 0.5% alginate mirrored the stiffness of normal colon tissues (0.936 kPa).^[^ ^32^ ^]^ For comparison, the compressive modulus of Geltrex was 0.125 kPa, making it 60 times and 7 times softer than CRC and normal colon tissues, respectively (Figure 4B).

We observed CEACAM5 expression in PDOs cultured under various setting, and compared them to those in tissues from the same patient (Figure 4C,D). CEACAM5 expression in Std‐PDOs was clearly lower than that in the tissues. Conversely, in the 1.5% alginate bath designed to mimic CRC tissue stiffness, CEACAM5 expression was comparable to that found in the original tissue, which agrees with our RNA‐seq findings (Figure 4C). As alginate concentration increased, *CEACAM5* mRNA expression also increased because of the increased confinement stress, emphasizing the importance of replicating physiologically relevant stress conditions to accurately model CRC progression and marker expression (Figure 4D).

Furthermore, we discovered that Eba‐PDOs accurately reflected CEACAM5 expression pattern in CRC tissues. Normally, CEA family proteins are expressed on apical surfaces of epithelial cells; however, in CRC, they are aberrantly directed to both apical and basolateral surfaces owing to a loss of cell polarity (Figure 4E,F). CEACAM5 on basolateral surfaces on CRC cells diminishes bonding within cells and between cells and collagen, thereby increasing the risk of metastasis.^[^ ^33^ ^]^ Confocal immunofluorescence analysis verified that Eba‐PDOs displayed atypical CEACAM5 expression on both apical and basolateral surfaces, resembling patterns observed in CRC tissues. In contrast, Std‐PDOs were unable to replicate this phenomenon (Figure 4E,F). These findings suggest that the stiff environment of the alginate bath used for Eba‐PDOs not only increases CEACAM5 expression but also disrupts cellular polarity, which is a key pathological feature of CRC.

### 2.5 Eba‐PDOs Capture Interpatient Heterogeneity in CEA Expression and Drug Sensitivity

**Figure 5:**
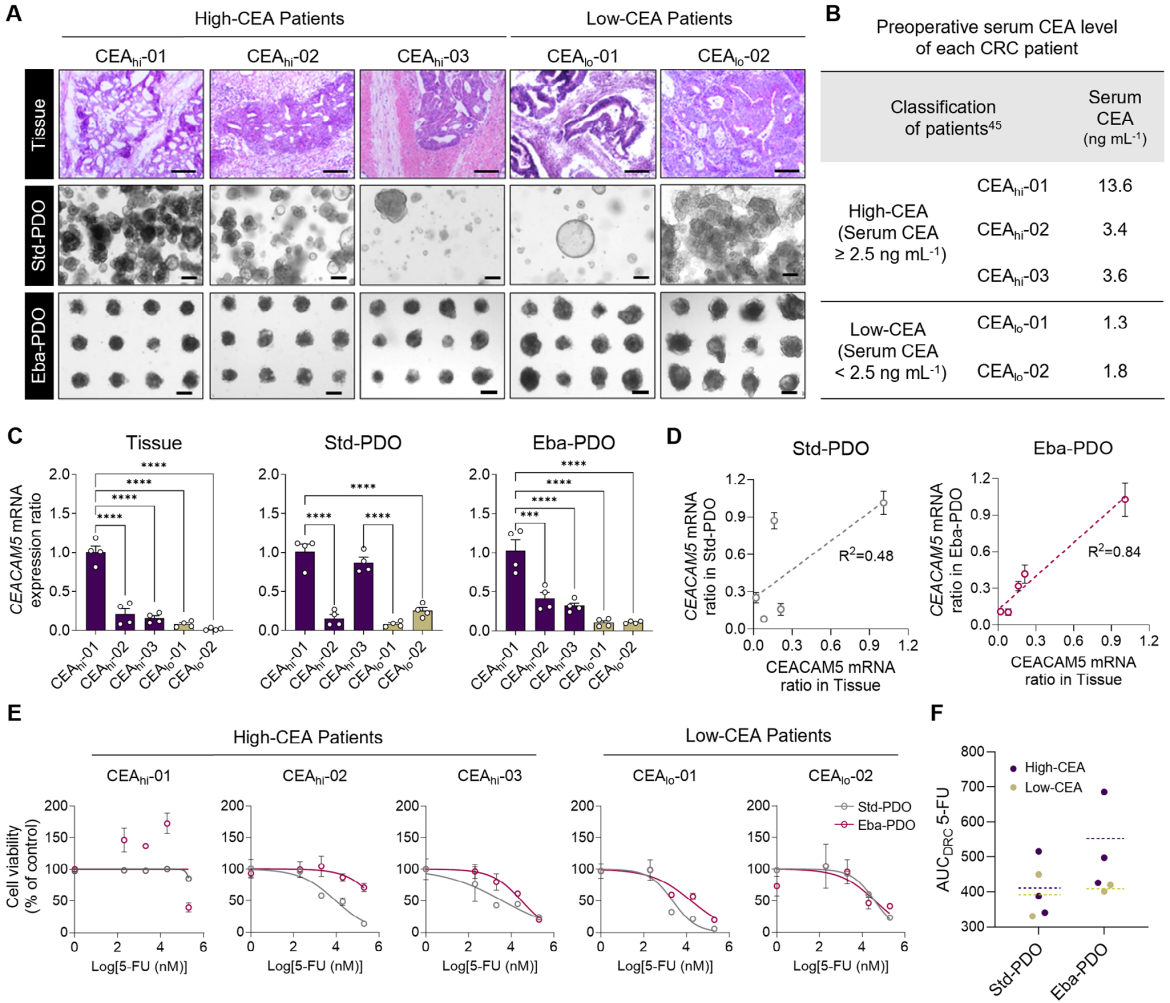
Interpatient variation in CEACAM5 levels and response to 5‐FU in PDOs. A) H&E stained sections of CRC tissue alongside bright field images of both Std‐ and Eba‐PDOs at 14 days. Scale bar = 100 µm (Tissue) and 200 µm (Std‐ and Eba‐PDOs). B) Preoperative serum CEACAM5 levels for each patient (CEA_hi_: High‐CEACAM5, CEA_lo_: Low‐CEACAM5). C) The ratio of *CEACAM5* mRNA expression in patient CRC tissues, Std‐PDOs, and Eba‐PDOs, compared to the CEA_hi_‐01 patient sample (*n* = 4 for all patients). D) Linear regression analysis between *CEACAM5* expression in PDOs and corresponding patient tissues (left: Std‐PDOs; right: Eba‐PDOs). E) Cell viability in Std‐ and Eba‐PDOs on day 6 post 5‐FU treatment at varying concentrations, assessed using the CellTiter‐Glo cell viability assay. Magenta indicates Eba‐PDOs and grey indicates Std‐PDOs (*n* = 3 for both PDO platforms). F) Individual area under curve (AUC) for Std‐ and Eba‐PDOs following 5‐FU treatment. The average for each group is shown with a dotted line (purple for High‐CEA; yellow for Low‐CEA). The results are presented as mean ± SEM. For statistical analysis, a one‐way analysis of variance and Tukey's test were performed (^***^ *p* < 0.001, ^****^ *p* < 0.0001, ns: non‐significant).

We further investigated the fidelity of Eba‐PDOs in accurately representing variability in CEACAM5 levels among individual patients. We generated Eba‐PDOs from five other patients and compared their characteristics with those of Std‐PDOs and actual CRC tissues (**Figure** **5**A). The five patients were categorized into two groups: High‐CEA (CEACAM5 level greater than 2.5 ng mL^−1^) and Low‐CEA (CEACAM5 level less than 2.5 ng mL^−1^), based on established clinical benchmarks for predicting survival and drug resistance from preoperative serum CEA levels^[^ ^34^ ^]^ (Figure 5B).

*CEACAM5* mRNA expression analysis in the original tissues revealed significant interpatient heterogeneity, varying across tumors from different patients (Figure 5C). This variability aligned with differences in patient serum CEACAM5 levels; tissues with higher serum CEACAM5 levels exhibited increased *CEACAM5* expression compared with those with lower CEA levels (Figure 5B,C). Eba‐PDOs demonstrated a strong correlation (correlation coefficient = 0.84) with the original tissue in *CEACAM5* mRNA expression. Eba‐PDOs derived from patient CEA_hi_‐01 displayed markedly elevated *CEACAM5* expression levels compared to tissues from patients CEA_hi_‐02 and CEA_hi_‐03 within the High‐CEA group, similar to observations in CRC tissues. Similarly, Eba‐PDOs from patients CEA_lo_‐01 and CEA_lo_‐02 showed lower expression levels than those from patients with High‐CEA levels (Figure 5C). In contrast, Std‐PDOs did not capture the *CEACAM5* expression variation observed among different patient tumors, showing a lower correlation coefficient of 0.48 (Figure 5D). *CEACAM5* expression was elevated in Eba‐PDOs across patient samples compared to Std‐PDOs, highlighting an improved ability to identify patient variability (Figure S10, Supporting Information). This is particularly important, as lower marker expression can obscure differences in CEACAM5‐associated drug responses and cellular behavior. The enhancement of *CEACAM5* expression in Eba‐PDOs, achieved in a 1.5% alginate bath (Figure 4D), could potentially be a key factor in making patient variability more apparent. These results emphasize the superior accuracy of Eba‐PDOs in capturing interpatient differences in *CEACAM5* expression.

Considering the extensive research demonstrating the association between serum CEACAM5 levels and cancer therapy resistance in patients with CRC,^[^ ^35^ ^]^ we investigated using embedded bioprinting to monitor the correlation between patient CEACAM5 levels and drug resistance. We evaluated the responsiveness of both PDO platforms to 5‐fluorouracil (5‐FU), a first‐line therapeutic agent for patients with CRC (Figure 5E). Patient CEA_hi_‐01, with clearly high CEACAM5 levels in tissue and serum, exhibited apparent resistance to 5‐FU on both PDO platforms. However, Std‐PDO cultures, which provide less dependable interpatient variability in *CEACAM5* expression, did not show an obserable disparity in 5‐FU sensitivity (AUC_DRC_5‐FU) between the High‐CEA and Low‐CEA PDOs (Figure 5F). A substantial difference was observed in average drug sensitivity between High‐CEA and Low‐CEA PDOs in Eba‐PDOs, supporting previous clinical findings,^[^ ^35^ ^]^ although further investigation involving a significantly larger number of PDOs is required to clarify this relationship. Our results indicate that Eba‐PDOs could offer a more accurate method for these analyses owing to their enhanced physiological resemblance to actual tissues compared to traditionl methods.^[^ ^36^ ^]^

### 2.6 Developing a Label‐ and Test‐Free Prediction Model Based on Eba‐PDO

Fully integrating PDOs into personalized medicine remains a significant challenge because extensive time and resources are required for toxicological testing and biochemical analyses. Therefore, integrating the biological relevance of PDOs with artificial intelligence for label and test‐free prediction of patient prognoses presents a promising strategy. Std‐PDOs exhibit clear morphological variations among patients (Figure 5A); however, the inherent heterogeneity in their size, shape, and internal structure complicates the development of predictive models based on imaging. In contrast, Eba‐PDOs demonstrate greater uniformity within samples from the same patient while still capturing distinct individual morphologies in tumor‐mimicking environments.

**Figure 6:**
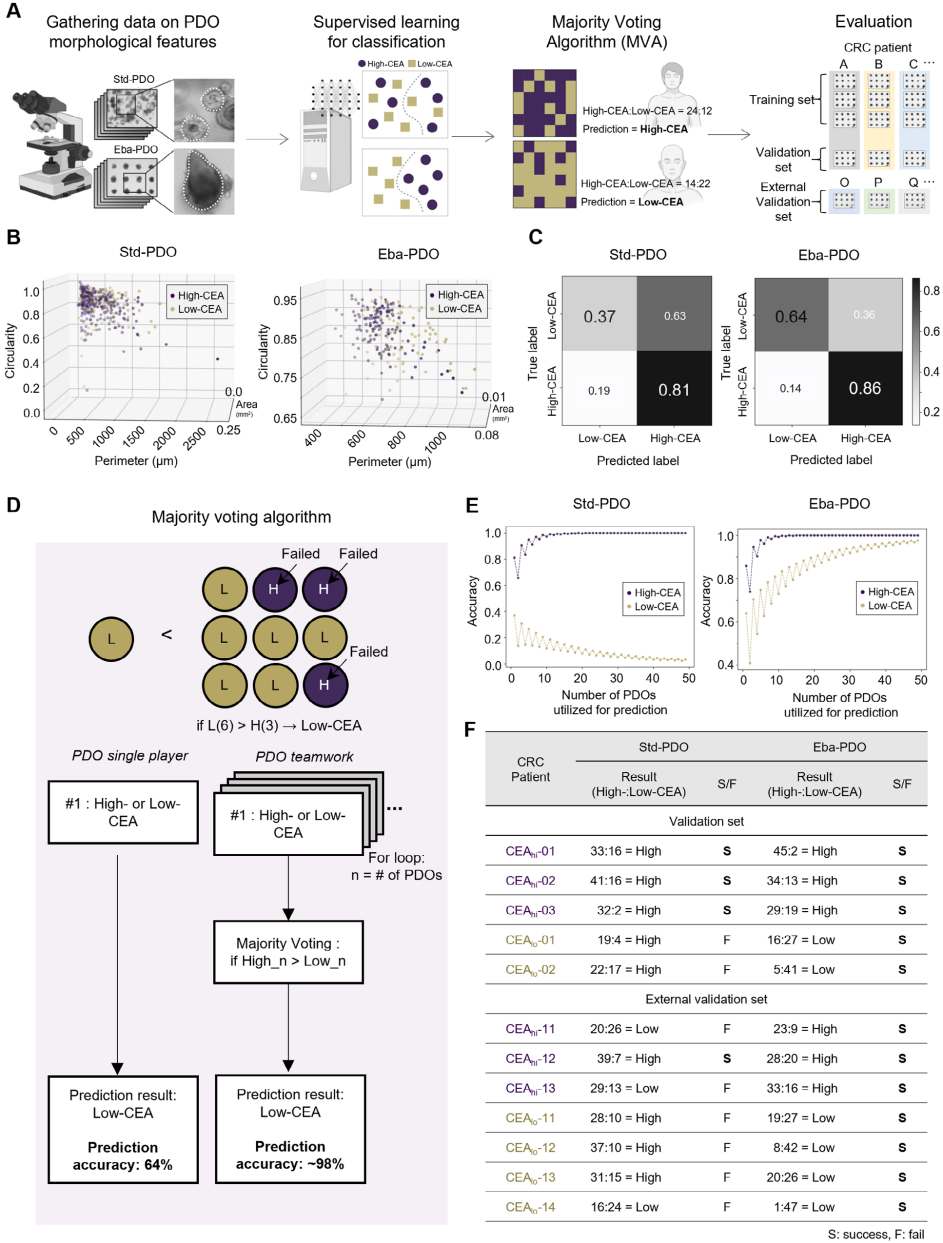
Eba‐PDO platform demonstrating the potential for label‐ and test‐free image‐based patient‐level prediction via supervised learning. A) Schematic illustrating the development of a High‐CEA/Low‐CEA prediction model using bright‐field images of PDO platforms via supervised learning. Morphological features such as area, perimeter, and circularity are extracted from the images of 177 Std‐PDOs and Eba‐PDOs from patients with CRC categorized as High‐CEA and Low‐CEA. These features were used to train Random Forest models. Predictions at the individual PDO level were then compiled using a majority voting algorithm (MVA) to establish patient‐level CEA predictions. The model's validation involved using a trained set and a different batch from the validation set of PDOs from the same patients. Evaluation was conducted using a test set of PDOs from different patients with CRC. B) The extracted morphological features are plotted on the 3D scale to show their distributions (*x*‐axis: perimeter (µm), *y*‐axis: area (mm^2^), *z*‐axis: circularity). Purple and yellow dots indicate High‐CEA and Low‐CEA, respectively. C) Confusion matrix for the Random Forest classifier displaying the prediction accuracy for High‐CEA/Low‐CEA in individual PDOs from both Std‐ and Eba‐PDO groups. D) Schematic illustration of the MVA to improve High‐CEA/Low‐CEA prediction accuracy via aggregating prediction results of individual PDOs. E) Simulation of patient‐level High‐CEA/Low‐CEA prediction accuracy using MVA, based on the number of PDOs utilized. F) Validation and evaluation of the MVA‐based prediction model using an untrained dataset from the same patients as those used in training, and an external validation set of PDOs from different patients with CRC, respectively. High: High‐CEA, Low: Low‐CEA. S: Success, F: Failure.

By leveraging Eba‐PDO uniformity, we explored the potential of applying supervised machine learning to develop a PDO image‐based prediction tool. We created a prediction tool to categorize PDOs based on High‐CEA and Low‐CEA levels, using only label‐free PDO bright‐field images for nondestructive PDO assessment and prediction. We compared the effectiveness between Std‐ and Eba‐PDOs (**Figure** **6**A). We compiled a data library from bright‐field images of 177 Std‐PDOs and Eba‐PDOs from five patients (CEA_hi_‐01∼03 and CEA_lo_‐01, 02) (Figure 5B) and analyzed attributes such as area, perimeter, and circularity (Figure 6B; Figure S11, Supporting Information). These data were used to train a Random Forest algorithm for binary classification of High‐CEA and Low‐CEA. The algorithm functions via generating and aggregating predictions from multiple decision trees to mitigate overfitting and improve generalization.^[^ ^37^ ^]^ It achieved an 86% success rate in identifying High‐CEA PDOs from Eba‐PDO samples but only a 64% accuracy for Low‐CEA PDOs when validated using trained images (training set). The Std‐PDOs, although similarly successful in classifying High‐CEA levels (81%), performed poorly at Low‐CEA levels (37%) (Figure 6C).

To enhance patient classification accuracy, we introduced the ‘majority voting algorithm (MVA)’ (Figure 6D). Unlike traditional methods that predict outcomes based on single PDO samples, MVA evaluates each PDO independently using a Random Forest algorithm and aggregates these multiple PDO evaluations to formulate a comprehensive patient‐level prediction. This approach was tested using theoretical simulations to determine variations in accuracy with the number of PDOs used during prediction (Figure 6E). Increasing the number of Eba‐PDOs consistently improved the accuracy of predictions for both High‐CEA and Low‐CEA classifications in MVA evaluations. For example, evaluating 50 Eba‐PDOs yielded 99% and 98% accuracy for High‐CEA and Low‐CEA patients, respectively. However, for Std‐PDOs, increasing the number of PDOs significantly reduced the Low‐CEA prediction accuracy to 3% when 50 PDOs were used, highlighting the importance of PDO uniformity in improving prediction models.

Our prediction models were initially validated using images of trained Std‐ and Eba‐PDOs (training set), confirming that both algorithms based on Std‐ and Eba‐PDOs could accurately classify patients (Figure 6F). It revealed that while the Random Forest algorithm occasionally failed to predict individual PDOs, the MVA consistently achieved high predictive accuracy for all tested patients. Our models were further validated with images from a new batch of Std‐ and Eba‐PDOs (validation set) from the same patients, confirming Eba‐PDOs' superior predictive ability (Figure 6F). This highlights both the reliability of the algorithm and the uniformity across the Eba‐PDO batches. Conversely, Std‐PDOs failed in identifying Low‐CEA patients, although they correctly predicted all High‐CEA patients, in agreement with the simulation results shown in Figure 6E. Further external validation was conducted with new PDOs derived from different seven patients—three High‐CEA (CEA_hi_‐11‐13) and four Low‐CEA (CEA_lo_‐11‐14)— with diverse subtypes, from pT2 to pT4, as well as from CMS1 to CMS3 subtypes (external validation set; Table S3 and Figure S12, Supporting Information) for unbiased evaluation of the model (Figure 6E). Notably, Eba‐PDO‐trained algorithm successfully predicted the classification for all seven patients, whereas the Std‐PDO‐trained model did not perform, even misclassifying the High‐CEA patients.

The enhanced prediction accuracy using Eba‐PDO‐based imaging training combined with the MVA is attributed to the high uniformity of Eba‐PDOs and their close resemblance to tumor tissues. This demonstrates the potential of the Eba‐PDO platform for broader applications in predicting patient outcomes, including sensitivity to specific drugs. This approach could enable clinicians to more precisely and quickly customize treatments based on the unique characteristics of each patient.

## 3 Conclusion

Overall, these results demonstrate that the embedded bioprinting approach used to create Eba‐PDOs, which markedly improves tissue resemblance and practical application. This innovative approach mimics the TME via replicating the specific matrix stiffness and hypoxic conditions, which promotes developing a 3D CRC model that more accurately mirrors the intrinsic and extrinsic features of the actual tumor tissue. We demonstrated that Eba‐PDOs captured the variability in CEACAM5 expression, a critical CRC marker, among patients. This variability correlates with patient categorization based on serum CEACAM5 levels and shows diverse responses to chemotherapy. In addition, Eba‐PDOs showed greater uniformity within the same patient, while retaining unique morphological differences between individuals; presents a more beneficial approach for developing an imaging‐based prediction model for clinical applications, which has previously proven to be challenging with traditional PDO cultures because of their significant heterogeneity.

To confirm the broader applicability of the Eba‐PDO platform, it will be necessary to expand the sample size to include a more diverse patient cohort in CRC and potentially other tumor types. Additionally, refining the predictive capabilities of Eba‐PDOs by incorporating a wider range of tumor features will be crucial. For example, Eba‐PDOs derived from a patient with a history of lymph node metastasis exhibited features indicative of tumor cell dissemination, including cell detachment and collective extrusion, which were absent in Std‐PDOs (Figure S13, Supporting Information). While preliminary, these findings suggest that supervised learning applied to Eba‐PDO data could be explored as a potential tool for assessing metastatic risk in patients in the future. Expanding these efforts will enhance the robustness and clinical relevance of the platform across diverse patient populations.

Additionally, integrating the cellular TME, including immune and endothelial cells will improve the precision of modeling patient‐specific tumor variations. We observed that Eba‐PDOs supported the infiltration of patient‐derived peripheral blood mononuclear cells (PBMCs) into PDOs (Figure S14, Supporting Information), highlighting their potential for modeling tumor‐immune interactions. Moreover, integrating vascular structures via bioprinting presents promising opportunities, as hypoxic conditions within Eba‐PDOs may drive angiogenesis,^[^ ^38^ ^]^ as suggested by enriched GO terms analysis. A bioprinted vascular network could enable more physiologically relevant nutrient exchange, oxygen gradients, immune infiltration, and drug diffusion dynamics.^[^ ^39^ ^]^ The variations in vascular structures formed within Eba‐PDOs could serve as morphological features for developing predictive algorithms to characterize patient‐specific tumor features.

Additionally, exploring more biocompatible materials, such as decellularized ECM, would also be beneficial for more accurately replicating the complex cellular environment of tumors. The unique features of this technology can be applied to various cancer models via integrating specific TME factors. This technology paves the way for highly personalized tumor models that could revolutionize personalized medicine, cancer drug development, and our understanding of tumor biology as influenced by the TME.

## 4 Experimental Section

### Tissues of Patients with CRC

All patient tissue samples and data used in this study were provided with approval from the Institutional Review Board (IRB, Approval No. 2019‐0340) of the Asan Medical Center. Additionally, the creation and use of PDOs were approved by IRB of UNIST (Approval No. UNISTIRB‐18‐49‐A). The research included participants diagnosed with CRC, and involved the use of resected CRC tissue segments larger than 1 cm^3^ for effiicent generation of CRC PDOs.

### Human CRC PDO Culture

The CRC PDOs were established according to the published protocol with slight modifications.^[^ ^40^ ^]^ The resected CRC segments measuring 1 cm^3^ were preserved in MACS Tissue storage solution (130‐100‐008, Miltenyi Biotec, North Rhine‐Westphalia, Germany) at 4 °C and used within 8 h of resection. To generate CRC PDOs, the resected CRC segments were washed twice in Dulbecco's phosphate‐buffered saline (DPBS) without Ca^2+^/Mg^2+^ containing 0.1 mg mL^−1^ Primocin (ant‐pm‐1, InvivoGen, CA, USA) and 5 µg mL^−1^ Plasmocin (ant‐mpp, InvivoGen). The washed CRC segments were then cut into small fragments (2–5 mm^2^), washed in DPBS, and placed in a digestion buffer consisting of basal medium (Advanced DMEM/F12; 12 634 028, Gibco, Thermo Fisher Scientific, MA, USA) containing 1.5 mg mL^−1^ type II Collagenase (17 101 015, Gibco), 20 µg mL^−1^ Hyaluronidase (H3506, Sigma‐Aldrich, MO, USA), and 10 µm Y27632 (1254, TOCRIS, Bristol, UK). The segments were incubated in a shaker at 37 °C for 1–3 h. At 1‐h intervals, the isolated CRC cell clumps were transferred to a new tube, and fresh digestion buffer was added to ensure high cell viability. The obtained cell clumps were treated with 5% fetal bovine serum (FBS; TMS‐013‐BKR, Merck, NJ, USA) to deactivate the enzymes. After centrifugation at 300 × g for 5 min at 4 °C, the pellet was resuspended in Geltrex (Invitrogen, CA, USA) and seeded in a nontreated 24‐well cell culture plate (20 µL droplet per well). After the Geltrex was solidified, ≈30 min post‐seeding, PDO culture medium^[^ ^40^ ^]^ (Table S2, Supporting Information) was added. At 10 days of culture, the CRC PDOs were dissociated with TrypLE Express (12 604 021, Gibco) via incubating for 5 min at 37 °C to create Eba‐PDOs.

### Preparation of PDO‐ and Bath‐Ink

For PDO printing, PDOs‐ and bath‐inks were developed via modifying the bio‐inks from our previous studies.^[^ ^41^ ^]^ The PDO‐ink was composed of Geltrex (A1413202, Gibco), which was used as an ECM to support the growth of the PDO, gelatin (G6144‐500G, Sigma), hyaluronic acid (53747‐10G, Sigma), and modified Eagle's minimum essential medium (MEM, 11‐095‐080, Gibco). Ink rheology was optimized for printing with gelatin and HA. HA (6 mg mL^−1^) was dissolved in MEM and incubated overnight at 37 °C. Gelatin (75 mg mL^−1^) was then gently stirred into the HA solution for 90 min. This 75 µL of gelatin/HA mixture was suspended with 7.5 × 10^7^ dissociated PDO cells in a 100 µL of calcium and magnesium‐free DPBS (Gibco). Then it is rapidly blended with 75 µL of Geltrex, loaded in a syringe, and placed in an ice immediately before printing.

The bath‐ink consisted of alginate (180947‐250G, Sigma), which served as a support bath during printing and for encapsulating the printed PDOs after post‐crosslinking, combined with gelatin (Sigma), HA (Sigma), and MEM (Gibco). The alginate (1.5% w/v) was gently dissolved with a gelatin (22.5 mg mL^−1^)/HA (3 mg mL^−1^) mixture to create the bath‐ink. Each ink was loaded into 1 mL Sterile 2‐Part Plastic Syringe with Luer Slip Tuberculin Tip (P‐158, Henke Sass Wolf, Tuttlingen, Germany) and cooled at 4 °C for 10 min. Before operating the printer, the syringes were installed in its dispensing module of the printer. A more detailed protocol can be found in the Supporting Information.

### Development of PBMC Co‐Cultivation Model with Std‐ and Eba‐PDOs

All blood samples used in this study were provided with approval from the IRB of UNIST (Approval No. UNISTIRB‐22‐37‐A). PBMCs were isolated using the density gradient medium Lymphoprep (0 7801, STEMCELL Technologies, Canada Inc.) as per manufacturer's instructions. Briefly, whole blood was mixed with DPBS containing 2% FBS at a 1:1 ratio. Next, 4 mL of Lymphoprep was pre‐loaded into a 15 mL conical tube, and 8 mL of diluted blood was layered on top of Lymphoprep carefully to minimize mixing of blood with Lymphoprep. The tube was then placed gently in a centrifuge (5810 R, Eppendorf) and centrifuged at 800 × g for 20 min. Finally, PBMC layer at the plasma:LymphoprepTM interface was collected without disturbing the erythrocye/granulocyte pellet.

To co‐culture PBMCs with each PDO model, on day 10 of culturing the unified PDOs formed through self‐assembly, 5 × 10^5^ PBMCs stained with 5 µm CellTracker Green CMFDA (C7025, invitrogen, CA, USA) for 15 min were washed with complete PDO culture media without CellTracker and then treated to Std‐PDOs and Eba‐PDOs. After two days of culturing with PBMCs, Both Std‐, and Eba‐PDOs were observed using the LSM980 confocal microscope (ZEISS, USA).

### Embedded PDO Printing to Generate Eba‐PDOs

A custom‐built bioprinting system^[^ ^42^ ^]^ was used for embedded PDO printing. The system included stages capable of controlled motion in the *x*‐, *y*‐, and *z*‐axes, multi‐head dispensers (SMP‐III mechanical dispenser, Musashi Engineering Inc., Tokyo, Japan) capable of simultaneously printing three inks, and an enclosure that could control temperature, humidity, and cleanliness.

For embedded PDO printing, an in situ spheroid printing process reported in our previous study was adapted.^[^ ^41b^ ^]^ Briefly, a 5 mm × 5 mm × 0.6 mm^3^ poly‐carprolacton (PCL, Polyscience, IL, USA) wall was printed at a continuous pressure of 150 kPa (nozzle diameter: 200 µm). The bath‐ink was discharged inside the wall to create a 3D alginate bath at a controlled extrusion rate of 0.046 mL s^−1^ (nozzle diameter: 300 µm). PDO‐ink was precisely extruded in the bath in a predefined pattern at a controlled extrusion rate of 0.01 µL s^−1^ for 1.5 s, forming 3D dots ≈200 µm in diameter. Following printing, the construct was placed in an ice bath for ≈30 min to achieve temporary thermal crosslinking. It was then further crosslinked in 40 mm CaCl_2_ solution (500 µL, diluted in PBS, C7902, Sigma) for ≈15 min. The fabricated constructs were transferred to nontreated 24‐well cell culture plates (500 µL culture medium per well) for subsequent experiments. A more detailed protocol can be found in the Supporting Information.

### Morphological and Histological Analyses

For paraffin sectioning, samples cultured for 14 days were fixed in 4% paraformaldehyde solution (prepared in 0.9% saline with 2.5 mm CaCl_2_, 30525‐89‐4, Junsei, Chemical Co.,Ltd., Tokyo, Japan). Fixed samples were rinsed with saline, dehydrated using a graded series of ethanol concentrations, treated with xylene (1330‐20‐7, Samchun, Chemical Co.,Ltd., Seoul, Korea), and embedded in paraffin overnight. Sections of 5 µm thickness were cut using a microtome(RM2125 RTS, Leica, Wetzlar, Germany). For cryosectioning, the harvested samples were washed, embedded in Tissue‐Tek O.C.T compound (4583, Sakura Finetek, Tokyo, Japan), and were frozen in a – 80 °C freezer. Frozen samples were sectioned at a thickness of 5 µm using a cryostat (CM1950, Leica). Sectioned samples were adhered to a glass microslide (NC1889570, Matsunami, Osaka, Japan).

Histological analysis was performed via staining the slides with haematoxylin and eosin (H & E; Sigma), as per manufacturer's instructions. The slides were immersed in haematoxylin for 7 min and in eosin for 2 min, then covered by a glass microslide before examination under a microscope (Leica).

For immunostaining, the frozen human tissue samples were fixed in 10% (v/v) neutral‐buffered formalin (F2013, BioSesang, Gyeonggi‐do, Korea) for 30 min at room temperature. The fixed human tissue samples were then washed for 30 min. Washed samples were blocked with 5% (w/v) bovine serum albumin (A9418, Sigma) in PBS for 1 h and immersed in primary antibody solution. Additionally, for 3D imaging of Std‐ and Eba‐PDOs, the whole sample was transferred to a microtube without sectioning. Before applying the primary antibody solution, the sample was permeabilized in DPBS with 0.1% Triton X‐100 (Sigma) and blocked for 1 h in 10% goat serum in DPBS with 0.1% Triton X‐100.

For each primary antibody solution, HIF1α (dilution ratio 1:100, NB100‐449, Novus, MI, USA), YAP/TAZ (1:200, D24E4, Cell Signaling Technology, MA, USA), collagen type 1 (1:200, ab6308, Abcam, Cambridge, UK), and CEACAM5 (1:200, #2383, Cell Signaling Technology) were prepared via diluting each dilution ratio in PBS. After washing, PDOs were stained with Goat Anti‐mouse IgG (H+L) Alexa Fluor 488 (1:200, A11001, Invitrogen) and Goat anti‐Rabbit IgG (H+L), and Alexa Fluor 488(1:200, A11034, Invitrogen) solutions. After additional washing, the PDOs were incubated with Hoechst 33 258 (1:1000, Sigma) solution or 4′,6‐diamidino‐2‐phenylindole (DAPI, 1 µg mL^−1^, Sigma) for staining the nucleus. Stained slides were observed using a fluorescence uplight microscope (DM4B, Leica microsystems). To quantify the fluorescence intensity, regions of interest were manually specified and analyzed using ImageJ software (NIH).

To observe the hypoxic status of the PDO, BioTracker 520 Green Hypoxia Dye at a final concentration of 5 µm was applied to Std‐ and Eba‐PDOs at one week of culture. After 1 h of incubation, the PDOs were washed with complete PDO culture media without BioTracker 520 Green Hypoxia Dye, followed by an additional 3 h incubation, and then observed using the LSM980 confocal microscope (ZEISS, USA).

### Quantitative Real‐Time Polymerase Chain Reaction (qRT‐PCR)

The mRNA expression levels were analyzed using qRT‐PCR. Total RNA of cells within a 3D printed construct was extracted using a AccuPrep Universal RNA Extraction Kit (BIONEER Corporation, Daejeon, Korea) and cDNA was synthesized using a AccuPower RocketScript Cycle RT PreMix (BIONEER Corporation) as per manufacturer's instructions. SYBR Green Real‐time PCR Master Mix (TOYOBO, Osaka, Japan) was used to perform qRT‐PCR in a CFX Connect Real‐Time PCR Detection System (Bio‐Rad Laboratories, CA, USA). The sequence of forward and reverse primers used for qRT‐PCR is follows; *YAP1* (5‘→3‘, Forward; TAGCCCTGCGTAGCCAGTTA, Reverse; TCATGCTTAGTCCACTGTCTGT), *MMP2* (5‘→3‘, Forward; GACGGTAAGGACGGACTC, Reverse; AAGTGGTCCGTGTGAAGT), *CDH1* (5‘→3‘, Forward; ATTTTTCCCTCGACACCCGAT, Reverse; TCCCAGGCGTAGACCAAGA), *CDH2* (5‘→3‘, Forward; TCAGGCGTCTGTAGAGGCTT, Reverse; ATGCACATCCTTCGATAAGACTG), *CEACAM5* (5‘→3‘, Forward; CTGTCCAATGACAACAGGACC, Reverse; ACGGTAATAGGTGTATGAGGGG), *GAPDH* (5‘→3‘, Forward; GGAGCGAGATCCCTCCAAAAT, Reverse; GGCTGTTGTCATACTTCTCATGG).

### Bulk RNA Sequencing

Total RNA was extracted from Std‐PDOs, Eba‐PDOs, and primary tissues from CRC patient CEA_hi_‐02 using TRIzol reagent (15 596 018, Invitrogen). Sequencing libraries were prepared using the SMARTer Stranded RNA Library Kit (TAKARA Bio. Inc., Shiga, Japan) and the samples were sequenced using the NovaSeq 6000 system (Illumina Inc., CA, USA). The obtained reads were aligned to the human genome hg38 using HISAT2 v.2.1.0, and transcript per kilobase million values were calculated using StringTie v.1.2.3b. Analysis was performed using iDEP.96.^[^ ^43^ ^]^

### Testing 5‐FU Response

The drug 5‐FU; F6627, Sigma–Aldrich) was prepared via dissolving it in dimethyl sulfoxide (DMSO, Sigma–Aldrich). The medium containing 5‐FU (dilution ratio 1:1000) was added to PDOs and cultured for 7 days. In the control group, PDOs were treated with DMSO (0.1% v/v) in the medium. After 3 days of drug treatment, the medium was replaced with fresh media. On the sixth day of drug treatment, the metabolic activity of each sample was measured using the CellTiter‐Glo 3D cell viability assay (Promega, USA), as per manufacturer‘s instructions. Briefly, after removing the old medium, 85 µL of the reagent mixed with the culture medium was added to each well. The mixture was gently agitated at 50 rpm for 30 min at room temperature on a rotator. Then, 100 µL of the mixture was extracted from each well, and luminescence was measured using a microplate reader (Synergy Neo2 Multi‐mode reader, BioTek, USA). Survival fraction of the samples was calculated using the following equation:

(1) $$\begin{array}{ccc} & & \text{Survival Fraction}\left(\%\right)\\ & & \;=\frac{\text{Luminescence intensity of drug treated group on day}\;6}{\text{Luminescence intensity of DMSO treated control on day}\;6}\times 100 \end{array}$$

### PDO Morphometric Analysis and Prediction

Bright field images of 177 Std‐ and Eba‐PDOs (day 14) from 5 patients (CEA_hi_‐01, CEA_hi_‐02, CEA_hi_‐03, CEA_lo_‐01, and CEA_lo_‐02) were randomly selected and subjected to morphological feature extraction using the ImageJ software (NIH). Metrics such as PDO area, perimeter, and circularity were quantified and systematically cataloged in a data library as a comma‐separated value file format. Each entry was labeled as High‐CEA or Low‐CEA based on the patient's clinical data (Figure 5B). The predictive models for High‐CEA and Low‐CEA classification were built utilizing labeled data library and a Random Forest algorithm, respectively, for Std‐ or Eba‐PDO. The Random Forest algorithm offers interpretability, computational efficiency, and predictability with smaller datasets, benefitted from ensemble learning.^[^ ^37^ ^]^ The framework for patient‐level CEA classification included two main components: first, single PDO‐level classification prediction from quantified morphological feature data of individual PDO images utilizing established Random Forest model; second, a MVA to aggregate PDO‐level predictions for improved decision‐making in patient‐level classification. For MVA, maximum 49 array of PDOs were utilized for collecting predictions. Outliers, lying beyond 1.5 times the interquartile range, ≈12.5% of the data at each end of the distribution, were removed from the dataset. Validation of the model involved using trained images of PDOs (training set) and untrained images from a new batch of PDOs (validation set) from the same patients (CEA_hi_‐01, CEA_hi_‐02, CEA_hi_‐03, CEA_lo_‐01, and CEA_lo_‐02). Additionally, the model was further evaluated using Std‐ or Eba‐PDO images from seven additional patients (CEA_hi_‐11‐13, CEA_lo_‐11‐14; Figure S12, Supporting Information).

The development of the predictive pipeline and Random Forest‐based binary classification model, along with its visualization, was executed in Python 4.3, referring to codes from the GitHub repository (park‐gb/wine‐classification‐rfc, 2020). In the code, NumPy and pandas were employed for data handling, warnings for managing messages, matplotlib.pyplot for visualization, pickle for object serialization, sklearn for various tasks including model selection, evaluation, and preprocessing, and specifically utilized RandomForestClassifier from sklearn.ensemble for building the classification model.

### Statistical Analysis

All data represent means (± SEM). Statistical analyses were performed with GraphPad Prism 10 (GraphPad Software), using a Student's *t*‐test to compare two data sets, and one‐way analysis of variance (ANOVA) followed by Tukey's multiple comparison test were used to compare different groups. Statistical significance was indicated by ^*^ *p* < 0.05; ^**^ *p* < 0.01; ^***^ *p* < 0.001; ^****^ *p* < 0.0001, and ns: non‐significant.

## Conflict of Interest

The authors declare no conflict of interest.

## Author Contributions

J.H. and H.‐J.J. contributed equally to this work as co‐first authors. J.H. and H.‐J.J. were involved in designing and conducting all experiments, as well as analyzing the data, working with S‐J.M., H‐W.K., and T‐E.P., who supervised the entire project. J.C., H.K., and J.I.P., assisted in performing experiments and developing the image‐based prediction model. T.K., K.M., S.S., K.P., J.‐H.S. and D.‐B.J. contributed to designing the experiments and interpreting the data. D.‐B.J., C.S.Y, and I.H.S. obtained CRC tissue samples and patient information according to the IRB‐approved protocol. The manuscript was prepared by J.H., H.‐J.J., H.‐W.K., and T.‐E.P., with input from all others.

## Supporting information



Supporting Information

Supporting TableS1

Supporting TableS2

## Data Availability

The data that support the findings of this study are available in the supplementary material of this article.
