# Supplementary material for: Bioprinted Patient‐Derived Organoid Arrays Capture Intrinsic and Extrinsic Tumor Features for Advanced Personalized Medicine
Source: Adv Sci (Weinh). 2025 Mar 28;12(20):2407871. doi: 10.1002/advs.202407871 (PMC12120747; doi:10.1002/advs.202407871)
Supplement: Supplementary file 1 — Supporting Information [file ADVS-12-2407871-s001.docx]

Supplementary Information for

**Bioprinted Patient-derived Organoid Arrays Capture Intrinsic and Extrinsic Tumour Features for Advanced Personalised Medicine**

Jonghyeuk Han^1,2,14^, Hye-Jin Jeong^1,3,14^, Jeonghan Choi^1^, Hyeonseo Kim^1^, Taejoon Kwon^1,4^, Kyungjae Myung^1,4^, Kyemyung Park^5^, Jung In Park^1^, Samuel Sánchez^6,7^, Deok-Beom Jung^8^, Chang Sik Yu^9^, In Ho Song^9^, Jin-Hyung Shim^10,11^, Seung-Jae Myung^8,12,13*^, Hyun-Wook Kang^1,*^, Tae-Eun Park^1,*^

^1^Department of Biomedical Engineering, Ulsan National Institute of Science and Technology (UNIST), Ulsan 44919, Republic of Korea

^2^Wallace H. Coulter Department of Biomedical Engineering, Emory University School of Medicine & Georgia Institute of Technology, Atlanta, GA 30332, USA

^3^Center for Genome Engineering, Institute for Basic Science, Daejeon 34126, Republic of Korea

^4^Center for Genomic Integrity, Institute for Basic Science, Ulsan 44919, Republic of Korea

^5^Graduate School of Health Science and Technology and Department of Biomedical Engineering, Ulsan National Institute of Science and Technology, Ulsan 44919, Republic of Korea

^6^Institute for Bioengineering of Catalonia (IBEC), The Barcelona Institute for Science and Technology (BIST), Barcelona 08028, Spain

^7^Catalan Institute for Research and Advanced Studies (ICREA), Barcelona 08010, Spain

^8^Digestive Diseases Research Center, University of Ulsan College of Medicine, Seoul 05505, Republic of Korea

^9^Division of Colon and Rectal Surgery, Department of Surgery, Asan Medical Center, University of Ulsan College of Medicine, Seoul 05505, Republic of Korea

^10^Research Institute, T&R Biofab Co. Ltd., Siheung 15111, Republic of Korea

^11^Department of Mechanical Engineering, Tech University of Korea, Siheung 15073, Republic of Korea

^12^Department of Gastroenterology, Asan Medical Center, University of Ulsan College of Medicine, Seoul 05505, Republic of Korea

^13^EDIS Biotech, Seoul 05505, Republic of Korea

^14^These authors contributed equally.

*Corresponding Authors:

Seung-Jae Myung, M.D., Ph.D., University of Ulsan College of Medicine (E-mail: sjmyung@amc.seoul.kr; Ph: +82-2-3010-3917)

Hyun-Wook Kang, Ph.D., UNIST (E-mail: hkang@unist.ac.kr; Ph: +82-52-217-2527)

Tae-Eun Park, Ph.D., UNIST (E-mail: tepark@unist.ac.kr; Ph: + 82-52-217-2614)


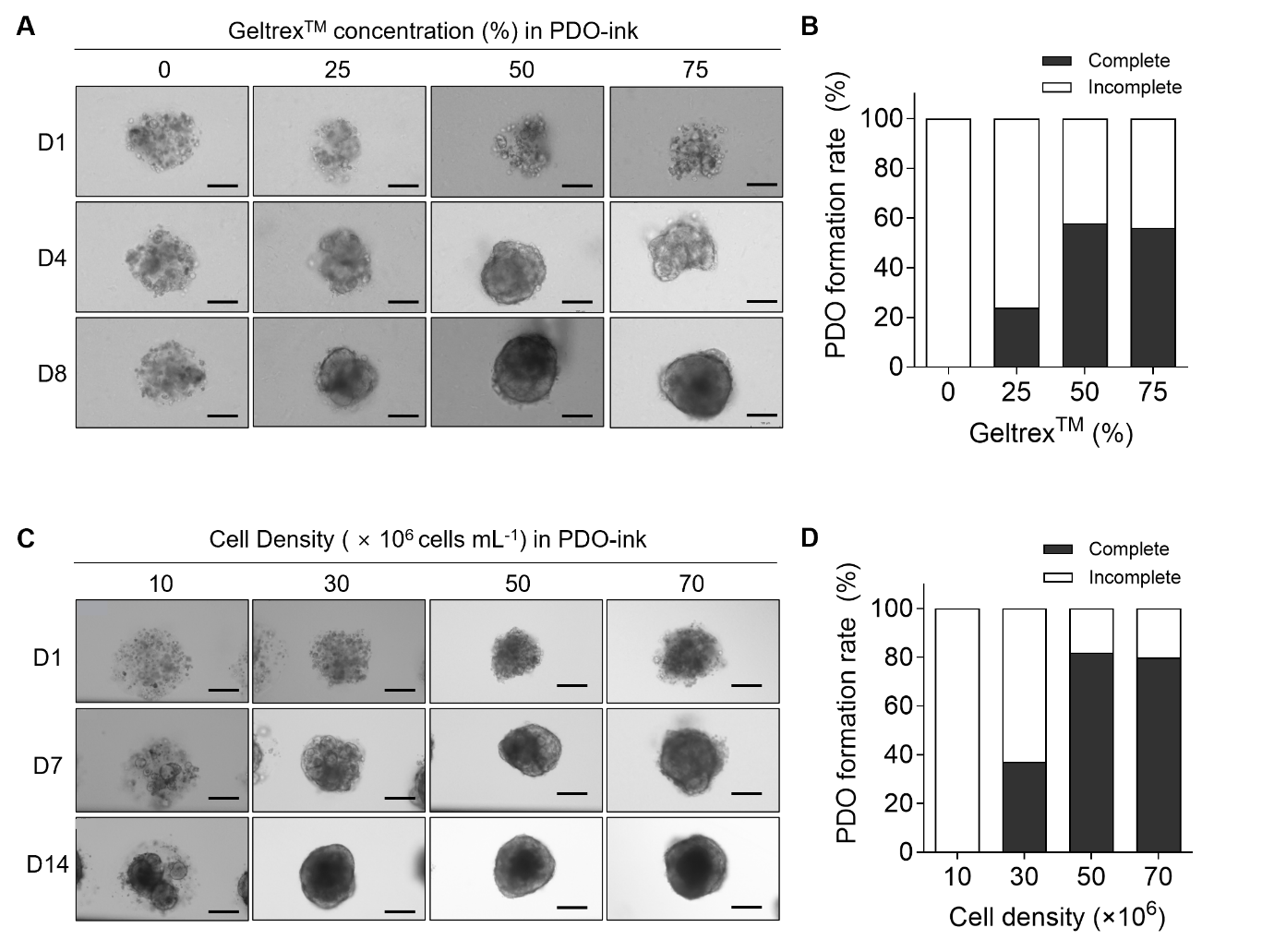


**Supplementary Fig. 1. Optimisation of PDO-Ink.** (A) Bright-field microscopy images showing an Eba-PDO formation across various concentrations of Geltrex^TM^ (0, 25, 50, and 75% v/v). Scale bar = 200 µm. (B) Graph depicting the time-dependent formation rate of unified Eba-PDO depending on the concentration of Geltrex^TM^. (C) Bright-field microscopy images showing Eba-PDO formation at different cell densities (10, 30, 50, and 70 × 10^6^ cells mL^-1^) in PDO-ink. Scale bar = 200 µm. (D) Graph showing the rate of unified Eba-PDO formation over time. Data were generated using PDOs from a CRC patient (CEA_lo_-11).

**
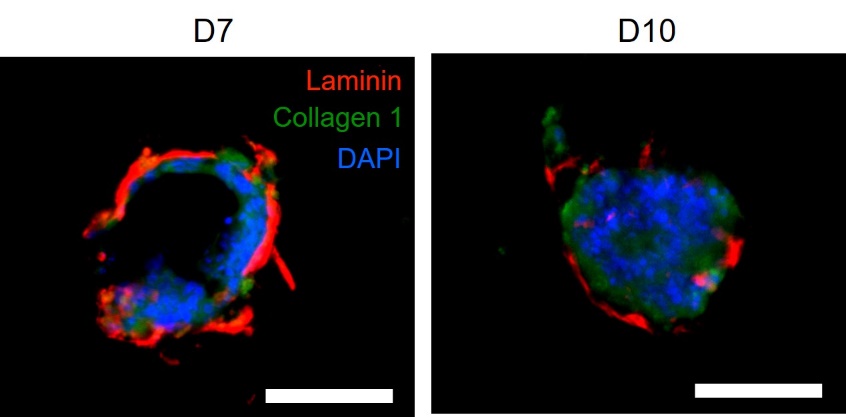
**

**Supplementary Fig. 2. ECM remodeling of Eba-PDO.** Immunofluorescence images showing ECM remodeling of an Eba-PDO overtime. Laminin (Red) protein expression decreased, and Collagen 1 (Green) expression elevated from day 7 to day 10. Scale bar = 200 µm.

**
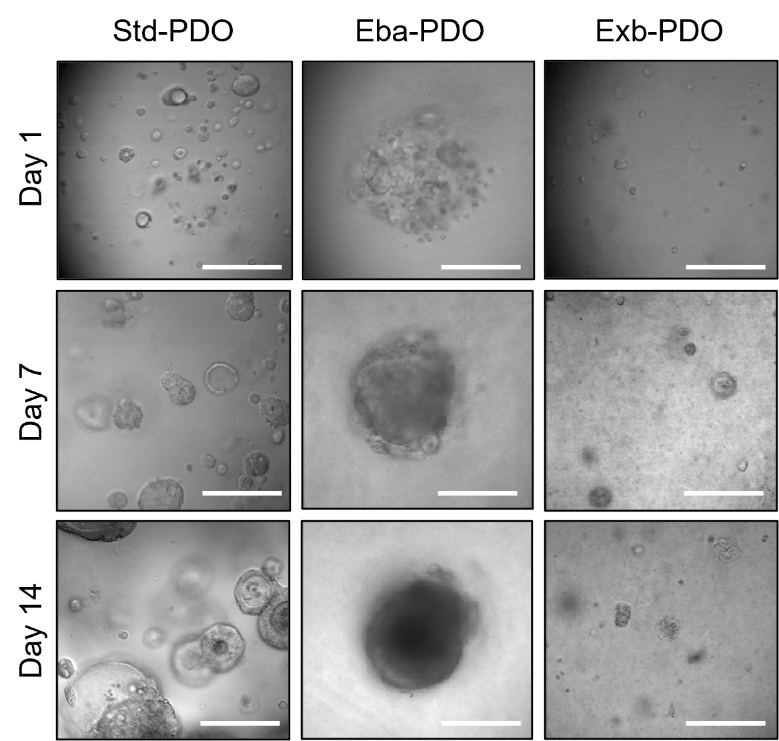
**

**Supplementary Fig. 3. Growth of PDOs in various platforms overtime.** Bright-field microscopy images illustrate successful growth of Std-PDO and Eba-PDO. In contrast, traditional extrusion-based bioprinting (Exb-PDO) using 1.5% alginate bioink with dissociated PDO cells suspended failed to support PDO growth on days 1, 7, and 14. Data were obtained using PDOs from a single CRC patient (CEA_lo_-11). Scale bar = 200 µm.

**
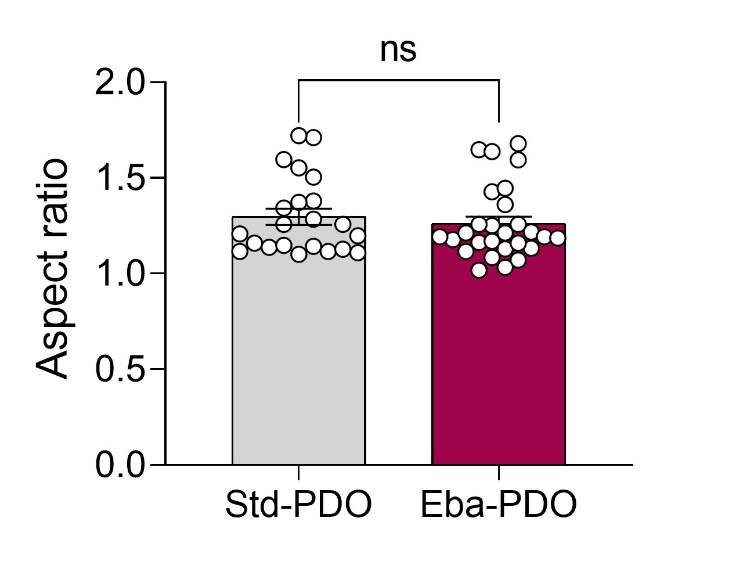
**

**Supplementary Fig. 4. Aspect ratio of Std- or Eba-PDOs.** Graph shows the aspect ratios of Std- and Eba-PDOs from organoids formed on day 14. Both Std- and Eba-PDOs exhibited a semi-spherical shape, possessing an aspect ratio of approximately 1.3. There were no statistically significant differences between the aspect ratios of Std- and Eba-PDOs. n > 22.


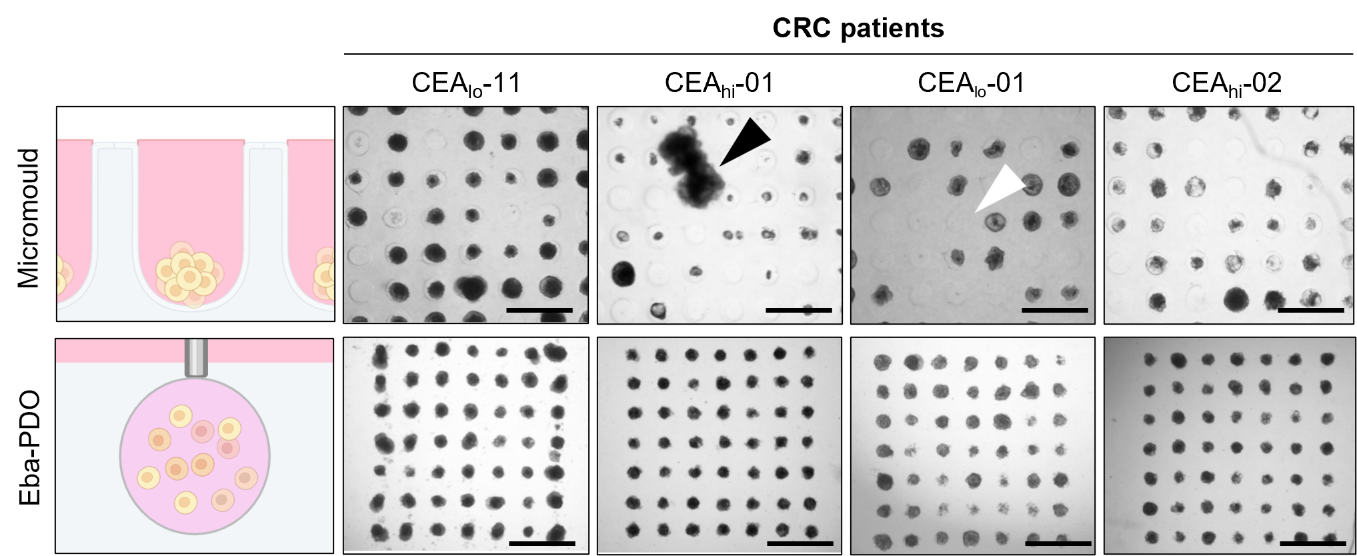


**Supplementary Fig. 5. Use of micromould and Eba-PDO culture platform for uniform PDO formation.** Bright-field microscopy images displaying the morphologies of PDOs from four CRC patients cultured on micromould (upper) and Eba-PDO platforms (lower). A black arrow points to aggregated PDO cells that are floating, while a white arrow highlights an empty well where PDO formation failed. Scale bar = 1 mm.


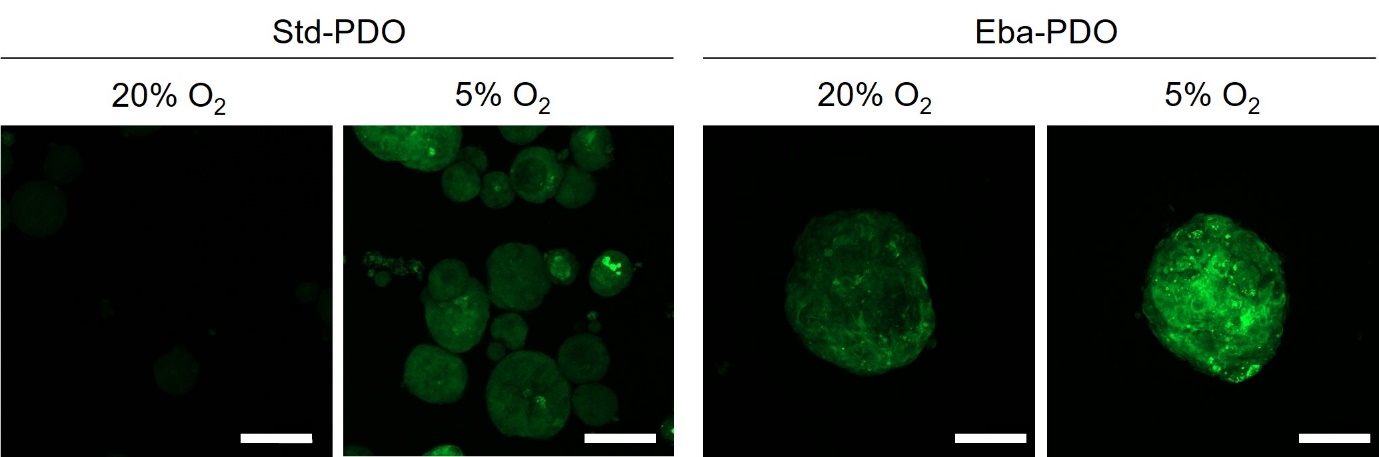


**Supplementary Fig. 6. Hypoxia detection in Std- or Eba-PDOs.** Immunofluorescence microscopy images displaying the results of BioTracker^TM^ 520 hypoxia dye staining in Std- (left) and Eba-PDOs (right) on day 7. PDOs were maintained either 5% (hypoxia) or 20% O_2_ (normoxia). Eba-PDOs displayed hypoxia cultured in 20% O_2_ conditions comparable to Std-PDO in 5% O2 hypoxia conditions (positive control). Scale bar = 100 µm.


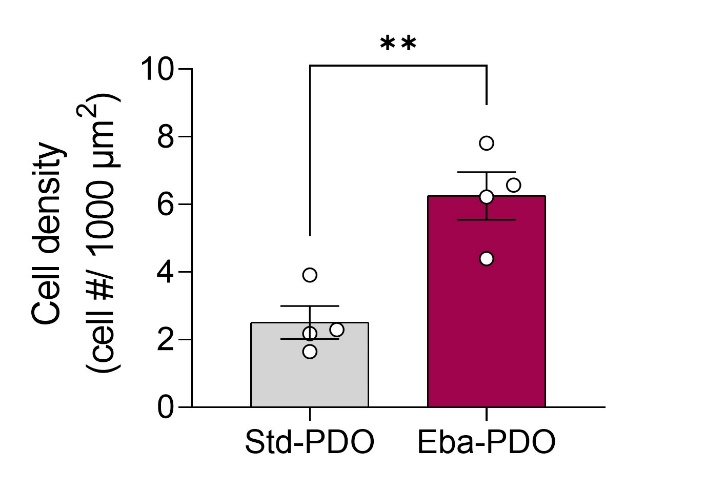


**Supplementary Fig. 7. Cell density in Std- or Eba-PDOs.** Graph shows cell numbers per unit area (1000 µm^2^) of Std- or Eba-PDOs on day 14. The area of each organoid was measured, and the cell number was counted in each organoid. Cell density was calculated by dividing the counted cell number by the organoid area. n = 4. ***P* < 0.001.


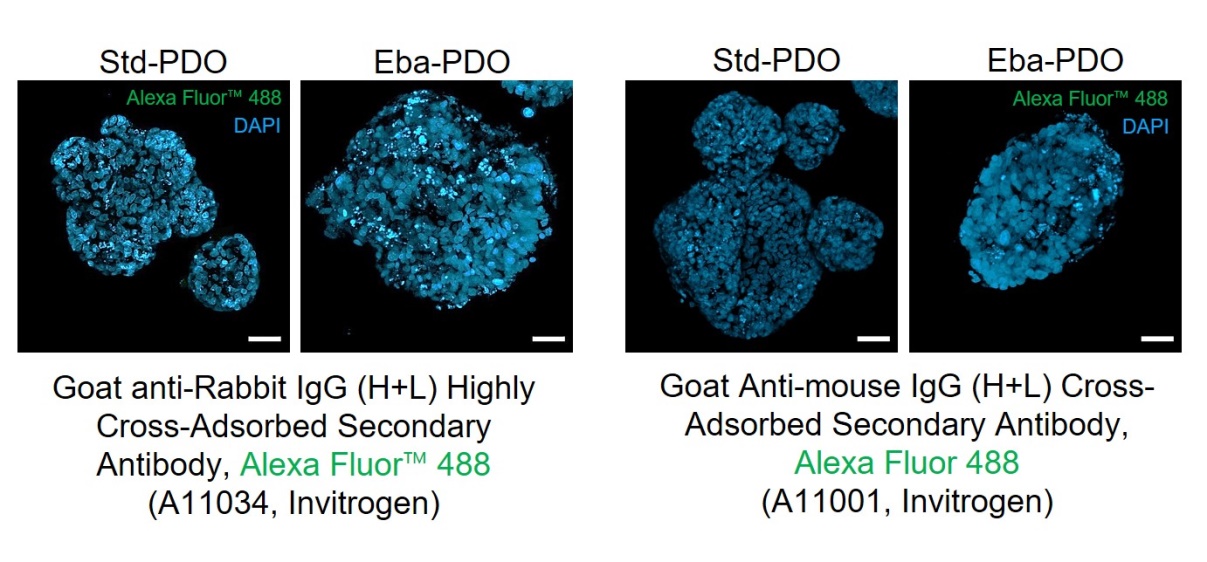


**Supplementary Fig. 8. Control images of PDOs stained with secondary antibodies only.** Immunofluorescence microscopy images showing the results of secondary antibody-Alexa Fluor™ 488 (Green) staining in Std- (left) and Eba-PDOs (right) on day 14. Staining was conducted without prior incubation with primary antibodies. No Alexa Fluor™ 488 signal was observed in either Std- or Eba-PDOs, confirming the absence of non-specific binding. Scale bar = 25 µm.


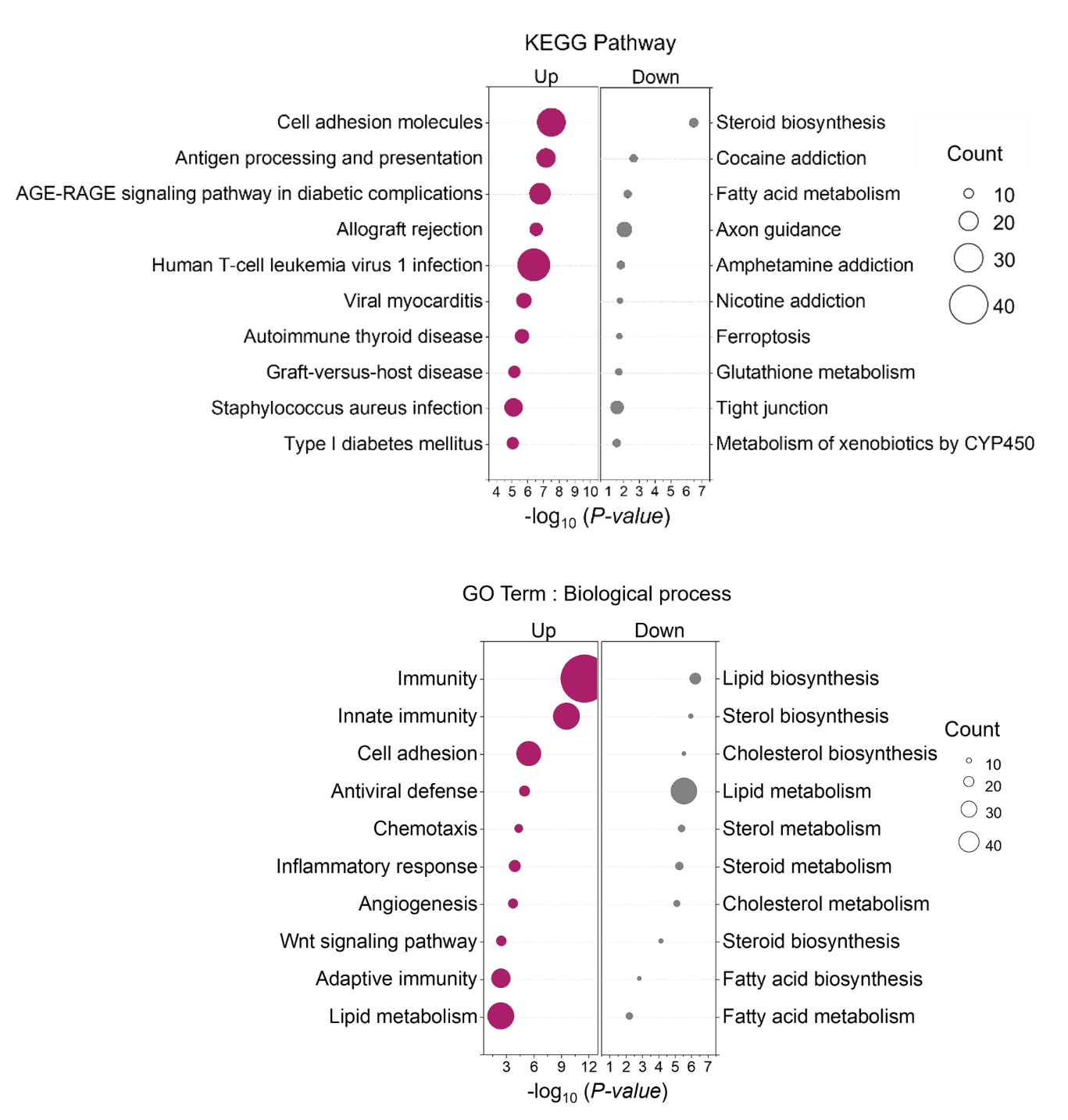


**Supplementary Fig. 9. Transcriptomic comparison of native CRC tissue (Tissue) compared to PDO platforms (Eba-PDO and Std-PDO).** Kyoto Encyclopedia of Genes and Genomes (KEGG) functional classification (top) and Gene Ontology (GO) term of biological process (bottom) showing the DEG that are upregulated and downregulated in CRC tissue compared to Std- and Eba-PDOs. The size of each dot represents the number of differential genes in the enrichment pathway. Data were obtained using PDOs from a single CRC patient (CEA_lo_-11).


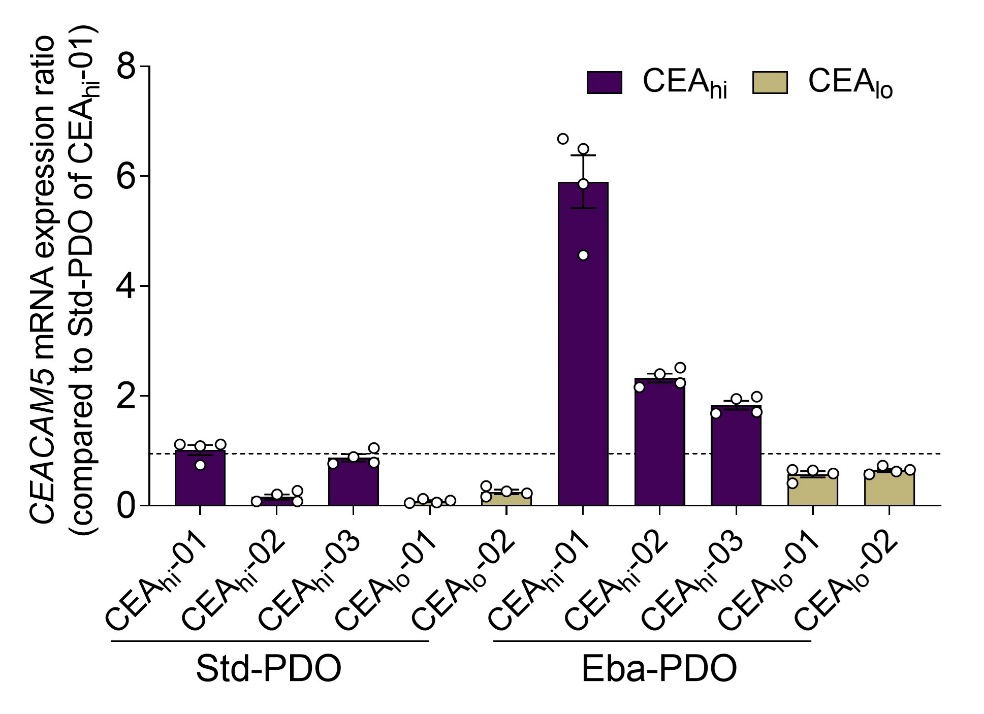


**Supplementary Fig. 10. CEACAM5 expression in Std- and Eba-PDOs across patients.** Measurement of *CEACAM5* mRNA expression in Std-PDOs and Eba-PDOs derived from five patients (CEA_hi_-01~03 (purple) and CEA_lo_-01, 02 (yellow)). CEA_hi_-01 was used as the normalisation standard, with its expression level set to 1 (indicated by the dotted line). Expression levels in other samples are presented as fold changes relative to CEA_hi_-01. Eba-PDOs consistently show higher *CEACAM5* expression compared to Std-PDOs across all patients.

**
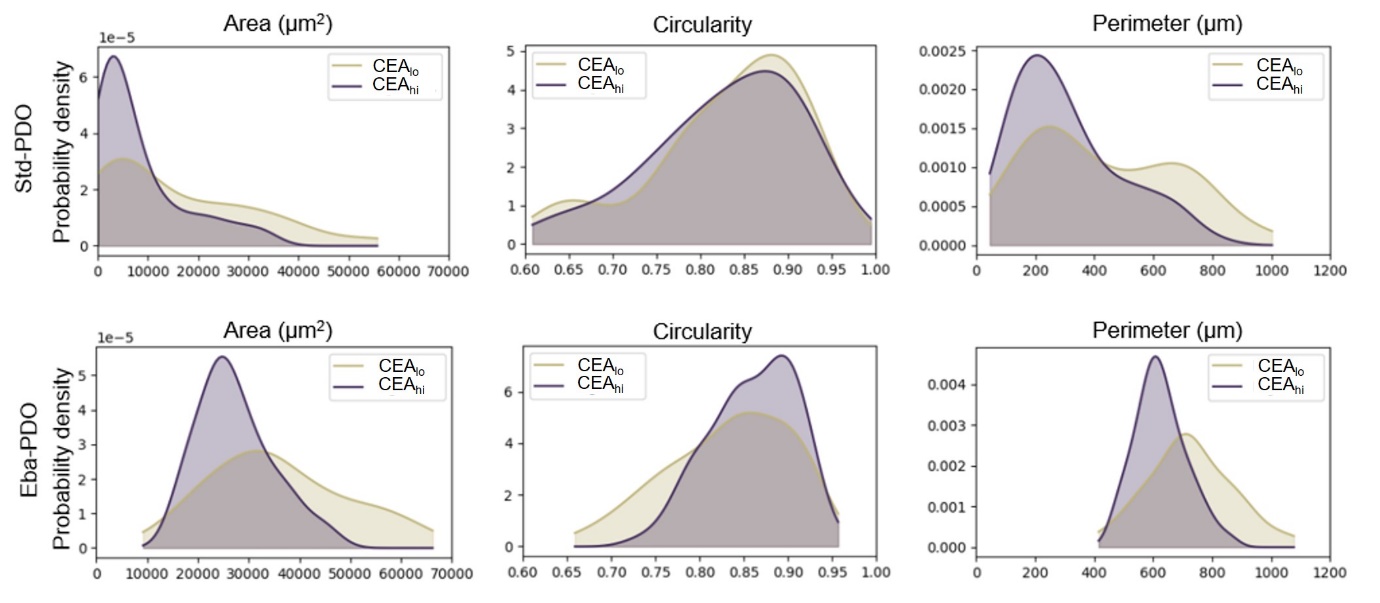
**

**Supplementary Fig. 11. Kernel density estimation plots depict the distribution of features in the dataset of Std- and Eba-PDOs.** Each subplot represents a different feature, with the kernel density estimation curves overlaid for the 'CEA_lo_' (yellow) and 'CEA_hi_' (purple) target groups, arranged in order of the importance of prediction. The shaded areas beneath the curves indicate the probability density for each group. The x-axis shows the range of values for each feature, while the y-axis shows the probability density. This visualisation offers a view of the data distribution and highlights potential predictive patterns.

**
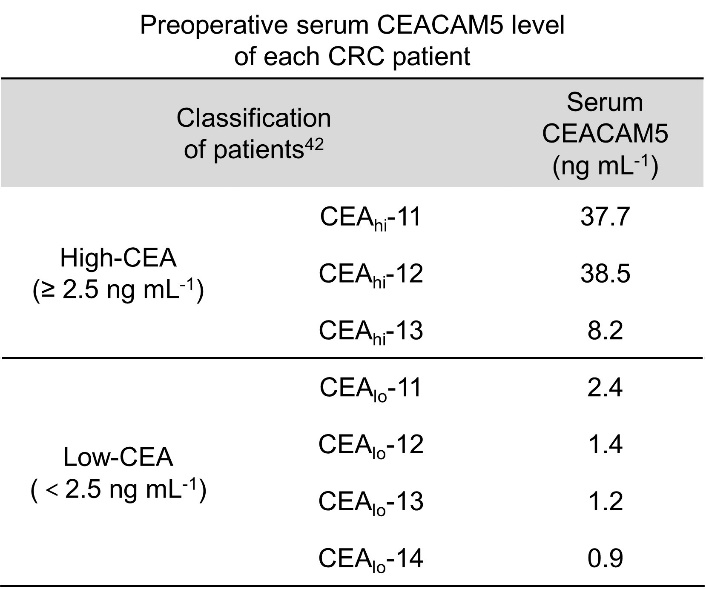
**

**Supplementary Fig. 12. Preoperative serum CEACAM5 levels for each patient used as a external validation set.** The PDOs from these patients served as a external validation set for evaluating our model predicting High-CEA and Low-CEA categorsation of CRC patient.

**
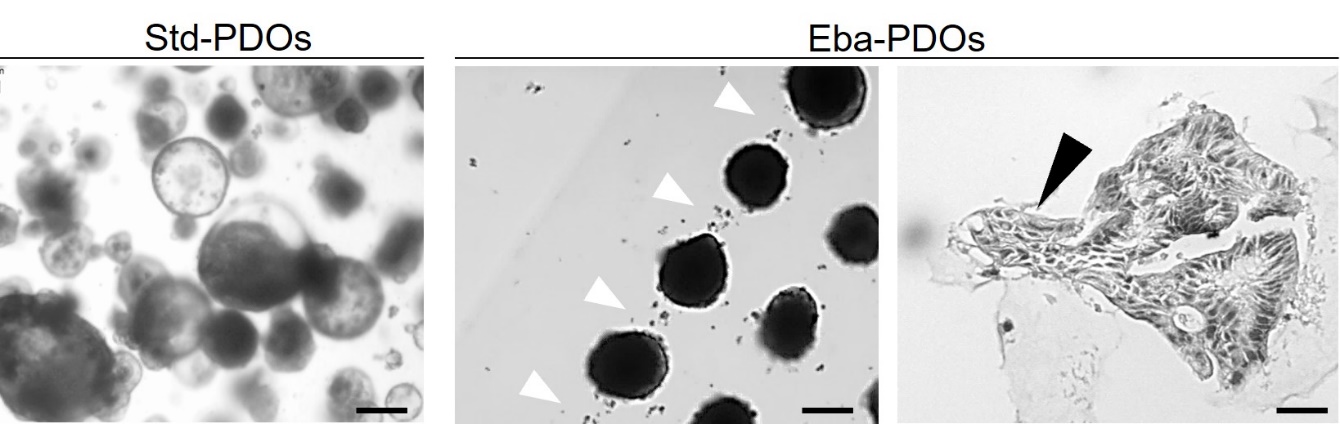
**

**Supplementary Fig. 13. Comparison of Std- and Eba-PDOs from a patient with a history of metastasis.** Bright field microscopic images of Std-PDOs (left panel) and Eba-PDOs (middle panel) and H&E-stained image of Eba-PDOs (right panel). Eba-PDOs show metastatic dissemination-like behaviors, which are absent in Std-PDOs. White arrows represent the cells detaching from tumours, and a black arrow represents collective extrusion of cells from Eba-PDOs. Data were obtained using PDOs derived from a single CRC patient (CEA_lo_-11) with lymph node metastasis. Scale bar = 250 µm for Std-PDOs (left panel) and Eba-PDOs (middle panel), and 25 µm for Eba-PDOs (right panel).

**
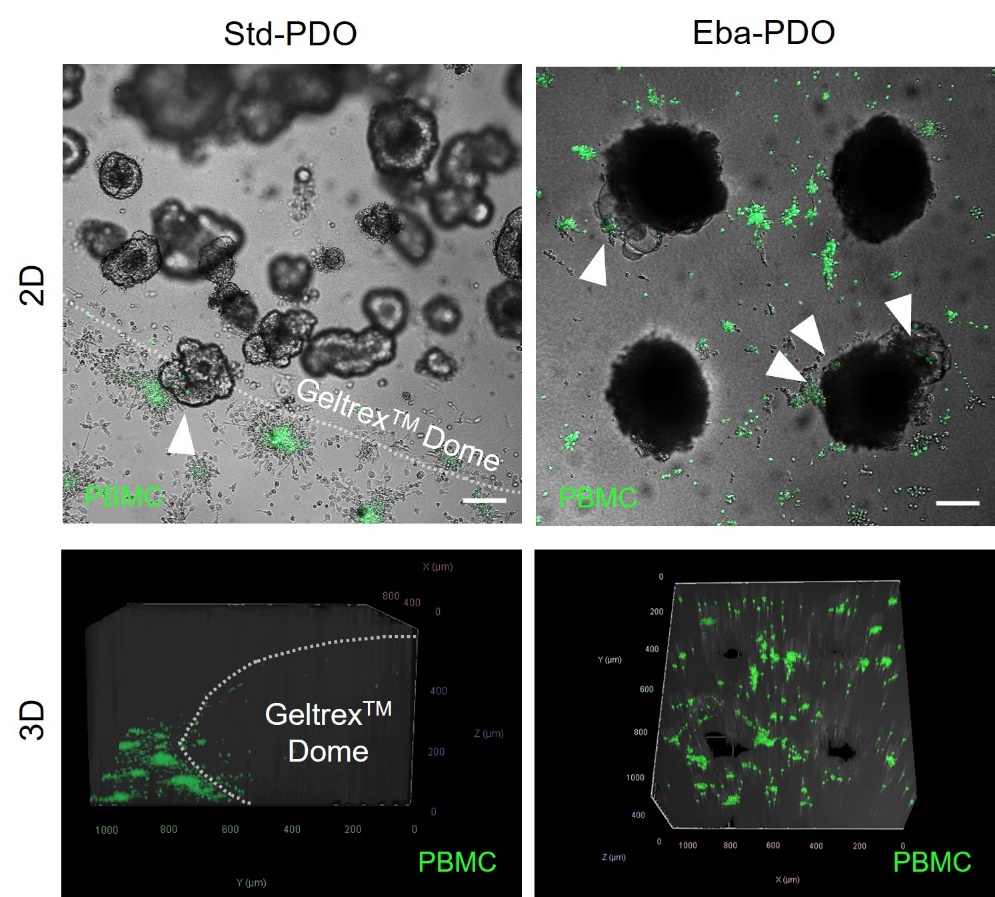
**

**Supplementary Fig. 14. PBMC co-cultivation model with Std- and Eba-PDOs.** 2D and 3D reconstructed confocal images display the distribution of PBMCs in the Std-PDO and Eba-PDO co-cultivation models. PBMCs are labeled green using CellTracker™ Green CMFDA. In the Std-PDO model, PBMCs showed minimal infiltration into the Geltrex™ dome and interact with the PDOs at the border-zone. In contrast, PBMCs infiltrate the alginate bath, showing more uniform distribution and interaction with Eba-PDOs within the same z-plane. Whited dotted lines indicate the outline of Geltrex^TM^ dome. White arrows indicate PDO-PBMC interactions. Scale bar = 100 µm.

**Supplementary Table 1. Transcriptomic comparison of Eba-PDOs compared to Std-PDOs.** The comparison of DEGs between Eba-PDOs and Std-PDOs focuses on genes that display a similar expression pattern between the Std-PDOs and the original tissue [log_2_ (fold change) > 1.0 (Eba-PDO versus Std-PDO), -1 < log_2_ (fold change) < 1.0 (Std-PDO versus Tissue), *P* < 0.05]. Gene Ontology (GO) term of biological process and Kyoto Encyclopaedia of Genes and Genomes (KEGG) indicates the significant enrichment of the DEG in biological process and pathways, respectively. Data were generated by analyzing PDOs from a CRC patient (CEA_lo_-11).

| **Category** | | **Term** | **Count** | **Gene ratio (%)** | ***P-value*** |
| --- | --- | --- | --- | --- | --- |
| **GO-**  **Biological process** | **UP** | Transcription | 65 | 9.94 | 5.21E-04 |
|  |  | Transcription regulation | 63 | 9.63 | 7.29E-04 |
|  |  | Cilium biogenesis/degradation | 10 | 1.53 | 0.00856 |
|  | **DOWN** | Lipid degradation | 8 | 1.31 | 0.00293 |
|  |  | Lipid metabolism | 26 | 4.26 | 0.00294 |
|  |  | Autophagy | 10 | 1.64 | 0.0039 |
|  |  | Cell adhesion | 18 | 2.95 | 0.01061 |
|  |  | Apoptosis | 19 | 3.11 | 0.01311 |
|  |  | Lipid transport | 8 | 1.31 | 0.04109 |
| **KEGG pathway** | **UP** | Herpes simplex virus 1 infection | 22 | 3.36 | 1.57E-04 |
|  |  | Purine metabolism | 7 | 1.07 | 0.02245 |
|  |  | Axon guidance | 8 | 1.22 | 0.03666 |
|  | **DOWN** | Systemic lupus erythematosus | 22 | 3.60 | 1.25E-11 |
|  |  | Alcoholism | 22 | 3.60 | 5.07E-09 |
|  |  | Neutrophil extracellular trap formation | 22 | 3.60 | 6.74E-09 |
|  |  | Viral carcinogenesis | 15 | 2.45 | 4.63E-04 |
|  |  | Necroptosis | 12 | 1.96 | 0.001755 |
|  |  | Autophagy - animal | 9 | 1.47 | 0.022158 |
|  |  | Mitophagy - animal | 6 | 0.98 | 0.031093 |
|  |  | Hippo signaling pathway  - multiple species | 4 | 0.65 | 0.033011 |
|  |  | Hippo signaling pathway | 9 | 1.47 | 0.038583 |

**Supplementary Table 2. Composition of human PDO culture medium**

| **No.** | **Component** | **Manufacturer** | **Catalog no.** |
| --- | --- | --- | --- |
| 1 | Advanced DMEM/F12 | Gibco | 12634028 |
| 2 | R-spondin1 conditioned media (10%, v/v) | Trevigen | 3710-001-K |
| 3 | Noggin (100 ng/mL) | Peprotech | 200-10C |
| 4 | Primocin (100 μg/mL) | InvivoGen | ant-pm-1 |
| 5 | Plasmocin (5 μg/mL) | InvivoGen | ant-mpp |
| 6 | GlutaMAX (2 mM) | Gibco | 35050061 |
| 7 | HEPES (10 mM) | Welgene | 15630106 |
| 8 | Nicotinamide (10 mM) | Sigma | N3376 |
| 9 | N-acetyl-L-cysteine (1.25 mM) | Sigma | A9165 |
| 10 | B27 supplement (1X) | Gibco | 17504044 |
| 11 | SB202190 (10 μM) | Bio-gems | 1523072 |
| 12 | Prostaglandin E2 (10 nM) | Peprotech | 3632464 |
| 13 | Animal-Free Recombinant  Human EGF (50 ng/mL) | Peprotech | AF-100-15 |
| 14 | A83-01 (500 nM) | TOCRIS | 2939 |
| 15 | Gastrin - [Leu15]-Gastrin I human (10 nM) | Sigma | G9145 |
| 16 | Y27632 (10 μM) | TOCRIS | 1254 |
| 17 | Fetal bovine serum (5%, v/v) | Merck | TMS-013-BKR |

**Supplementary Table 3. Clinical characteristics of patients from whom CRC PDOs were derived for MVA prediction**

**
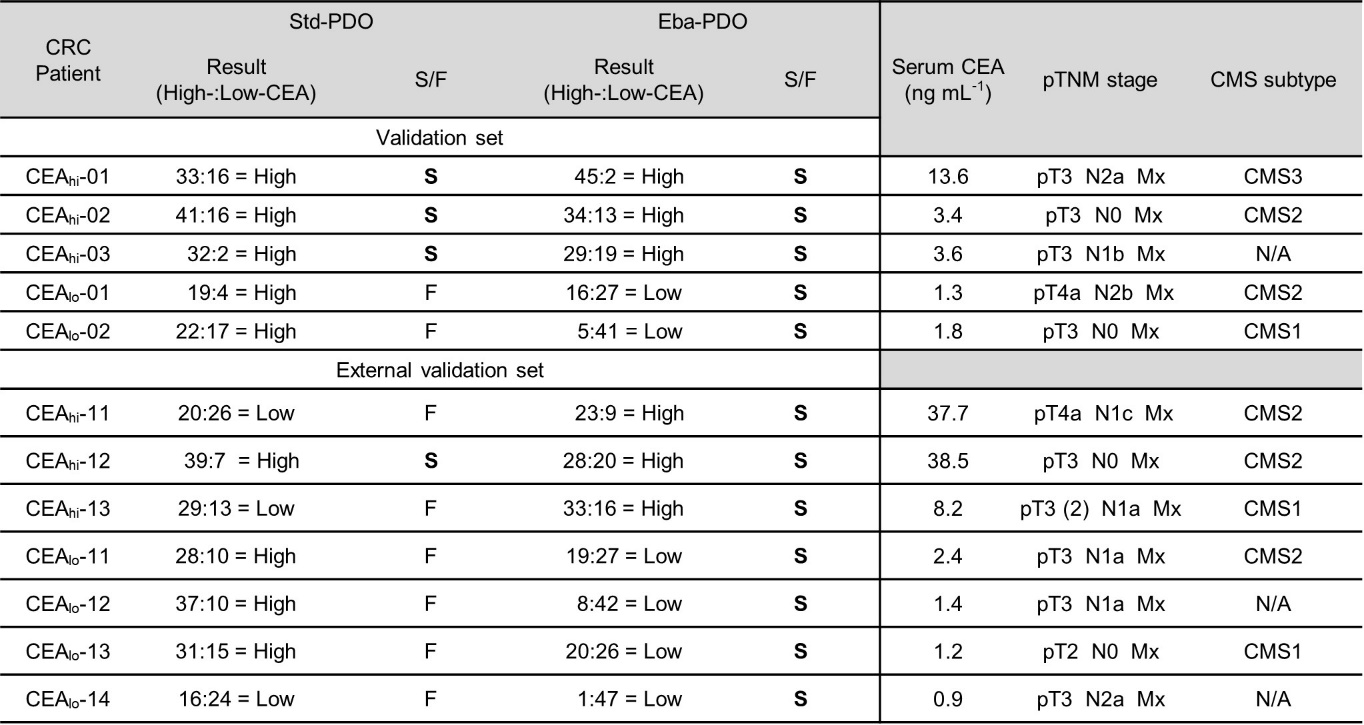
**

Supplementary Method for

**Bioprinted Patient-derived Organoid Arrays Capture Intrinsic and Extrinsic Tumour Features for Advanced Personalised Medicine**

Jonghyeuk Han^1,2,14^, Hye-Jin Jeong^1,3,14^, Jeonghan Choi^1^, Hyeonseo Kim^1^, Taejoon Kwon^1,4^, Kyungjae Myung^1,4^, Kyemyung Park^5^, Jung In Park^1^, Samuel Sánchez^6,7^, Deok-Beom Jung^8^, Chang Sik Yu^9^, In Ho Song^9^, Jin-Hyung Shim^10,11^, Seung-Jae Myung^8,12,13*^, Hyun-Wook Kang^1,*^, Tae-Eun Park^1,*^

^1^Department of Biomedical Engineering, Ulsan National Institute of Science and Technology (UNIST), Ulsan 44919, Republic of Korea

^2^Wallace H. Coulter Department of Biomedical Engineering, Emory University School of Medicine & Georgia Institute of Technology, Atlanta, GA 30332, USA

^3^Center for Genome Engineering, Institute for Basic Science, Daejeon 34126, Republic of Korea

^4^Center for Genomic Integrity, Institute for Basic Science, Ulsan 44919, Republic of Korea

^5^Graduate School of Health Science and Technology and Department of Biomedical Engineering, Ulsan National Institute of Science and Technology, Ulsan 44919, Republic of Korea

^6^Institute for Bioengineering of Catalonia (IBEC), The Barcelona Institute for Science and Technology (BIST), Barcelona 08028, Spain

^7^Catalan Institute for Research and Advanced Studies (ICREA), Barcelona 08010, Spain

^8^Digestive Diseases Research Center, University of Ulsan College of Medicine, Seoul 05505, Republic of Korea

^9^Division of Colon and Rectal Surgery, Department of Surgery, Asan Medical Center, University of Ulsan College of Medicine, Seoul 05505, Republic of Korea

^10^Research Institute, T&R Biofab Co. Ltd., Siheung 15111, Republic of Korea

^11^Department of Mechanical Engineering, Tech University of Korea, Siheung 15073, Republic of Korea

^12^Department of Gastroenterology, Asan Medical Center, University of Ulsan College of Medicine, Seoul 05505, Republic of Korea

^13^EDIS Biotech, Seoul 05505, Republic of Korea

^14^These authors contributed equally.

*Corresponding Authors:

Seung-Jae Myung, M.D., Ph.D., University of Ulsan College of Medicine (E-mail: sjmyung@amc.seoul.kr; Ph: +82-2-3010-3917)

Hyun-Wook Kang, Ph.D., UNIST (E-mail: hkang@unist.ac.kr; Ph: +82-52-217-2527)

Tae-Eun Park, Ph.D., UNIST (E-mail: tepark@unist.ac.kr; Ph: + 82-52-217-2614)

**Generation and Culture of Embedded Bioprinting-enabled Arrayed Patient-Derived Organoids (Eba-PDOs)**

**Abstract**

This protocol provides a comprehensive guide for the generation, culture, and 3D bioprinting of human colorectal cancer (CRC) patient-derived organoids (PDOs). It covers the detailed steps involved in tissue sample preparation, PDO culture, and the bioprinting of Eba-PDOs using custom bio-inks and a specialized bioprinting system. The protocol adheres to approvals by the Institutional Review Board of the Asan Medical Center (Approval No. 2019-0340) and UNIST (Approval No. UNISTIRB-18-49-A).

**Reagent**

| **Reagent** | **Supplier** | **Catalog Number** |
| --- | --- | --- |
| MACS® Tissue Storage Solution | Miltenyi Biotec | 130-100-008 |
| Dulbecco's Phosphate-Buffered Saline (DPBS) | Gibco | without Ca²⁺/Mg²⁺ |
| Primocin | InvivoGen | ant-pm-1 |
| Plasmocin | InvivoGen | ant-mpp |
| Advanced DMEM/F12 | Gibco | 12634028 |
| Type II Collagenase | Gibco | 17101015 |
| Hyaluronidase | Sigma-Aldrich | H3506 |
| Y27632 | TOCRIS | 1254 |
| Fetal Bovine Serum (FBS) | Merck | TMS-013-BKR |
| Geltrex™ | Invitrogen | A1413202 |
| TrypLE Express | Gibco | 12604021 |
| Gelatin | Sigma | G6144-500G |
| Hyaluronic Acid (HA) | Sigma | 53747-10G |
| Modified Eagle's Minimum Essential Medium (MEM) | Gibco | 11-095-080 |
| Alginate | Sigma | 180947-250G |
| Polycaprolactone (PCL) | Polyscience | N/A |
| Calcium Chloride (CaCl₂) | Sigma | C7902 |


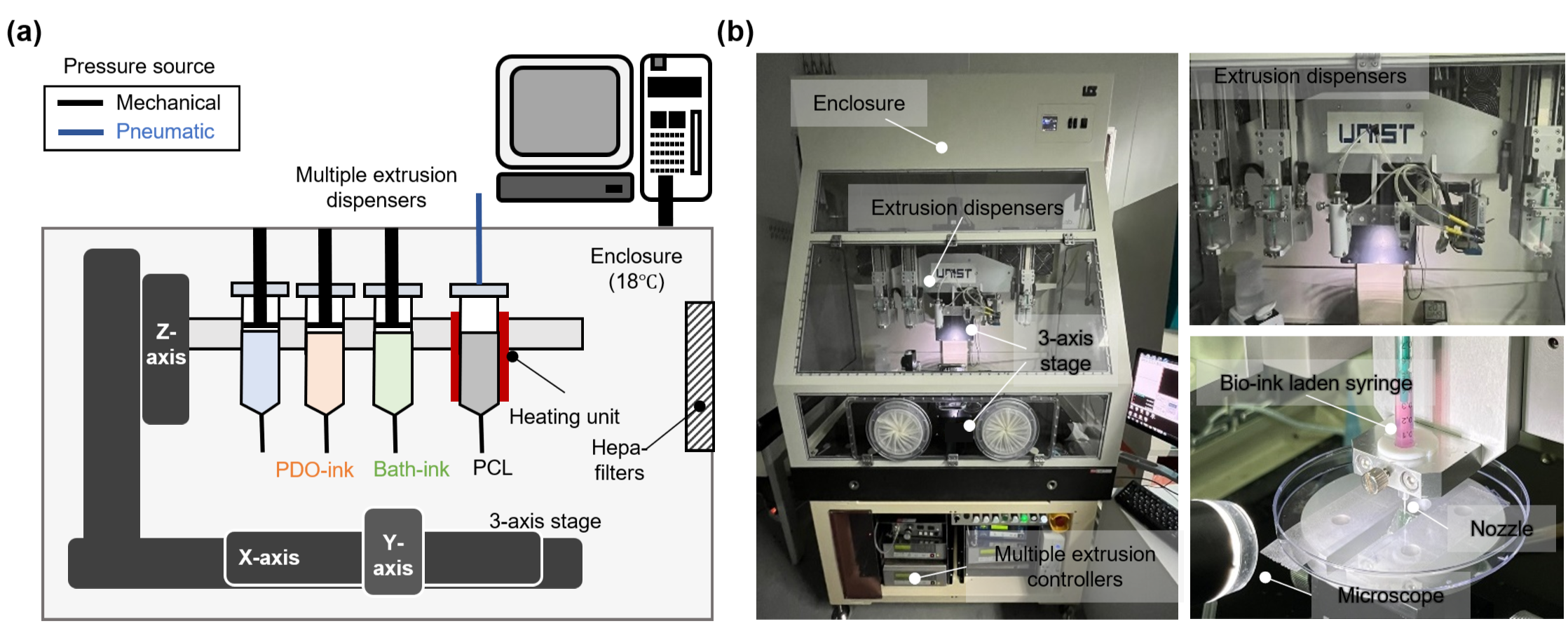
**Equipment**

**Figure 1. Extrusion based bioprinting system.** 3D bioprinting system was equipped with multiple extrusion dispensers to fabricate Eba-PDO model. **(a)** PDO-ink and Bath-ink were extruded by SMP III mechanical dispenser. Polycaprolactone (PCL) was melted by heating unit and extruded by pneumatic dispenser. **(b)** The bioprinting system was composed by enclosure system and 3-axis stage. Bio-ink laden syringe was applied to the dispenser with micro-sized nozzle. The printing process was observed by microscope.

| **Equipment** | **Description/Specifications** |
| --- | --- |
| Bioprinting system | SMP-III mechanical dispenser, Musashi Engineering Inc. (Figure 1) |
| 24-well cell culture plates | Non-treated, for PDO seeding and culture |
| Sterile 2-Part Plastic Syringe | Henke Sass Wolf, P-158 |
| Centrifuge | For pelleting cells |
| Shaker | For gentle mixing during incubation |
| Incubator | Set at 37 °C for optimal cell culture conditions |
| Ice bath | For cooling bio-inks and maintaining viscosity |
| Controlled motion stages and multi-head dispensers | Precise movement in x, y, z axes for bioprinting |
| PCL nozzle | SHN-0.2N, sterilized with 70% ethanol |
| PDO-ink nozzle | SPN-0.12-0.65L, sterilized for precise cell extrusion |
| Bath-ink nozzle | SHN-0.3N, for uniform bath ink dispensing |

**Procedures**

**1. Tissue Preparation and CRC PDO Culture**

1. Tissue Collection: Collect resected CRC tissue segments (>1 cm³), preserve in MACS® Tissue Storage Solution at 4 °C. Use within 8 hours.
2. Washing: Wash segments twice in DPBS containing 0.1 mg/mL Primocin and 5 μg/mL Plasmocin.
3. Digestion: Cut segments into 2–5 mm² fragments; incubate in digestion buffer (Advanced DMEM/F12, 1.5 mg/mL Collagenase, 20 μg/mL Hyaluronidase, 10 μM Y27632) at 37 °C for 1–3 hours with gentle shaking.
4. Enzyme Deactivation: At hourly intervals, transfer cell clumps to a fresh tube; deactivate enzymes with 5% FBS.
5. Centrifugation & Resuspension: Centrifuge at 300 × g for 5 min at 4 °C; resuspend the cell pellet in Geltrex™ and seed in 24-well plates (20 µL droplets).
6. Culturing: After solidification of Geltrex™, add PDO culture medium. Incubate for 3 days, then dissociate and replate to expand. Repeat as needed.

**2. Preparation of Bath-Ink**

1. Solution Preparation: Dissolve HA (3 mg/mL) in MEM; rotate overnight at 37°C. Add gelatin (22.5 mg/mL) and alginate (1.5% w/v). Mix gently at 37°C for 90 minutes.
2. Sterilization: Use a 0.45 μm filter for sterile filtration, ensuring sterility under laminar flow.
3. Cooling: Load the bath-ink into sterile syringes and place in an ice bath to maintain desired viscosity during printing.

**3. Preparation of PDO-Ink**

1. Dissolve HA: Dissolve HA in MEM (6 mg/mL); rotate overnight at 37°C until fully dissolved.
2.
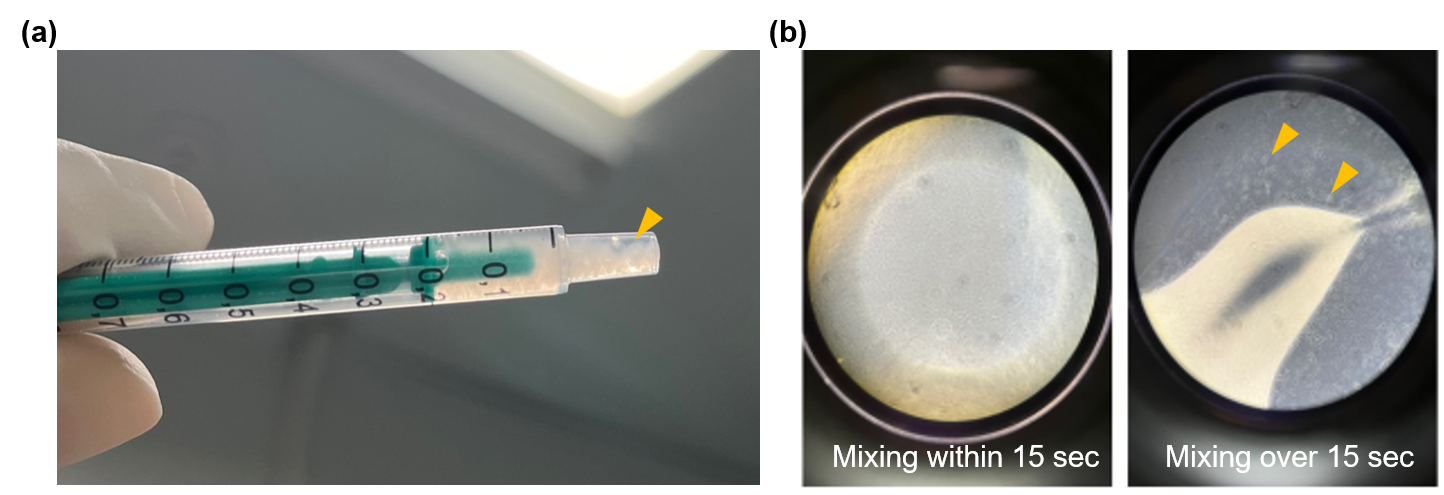
Gelatin/HA Mixture: Add gelatin (75 mg/mL) to HA-MEM solution. Rotate at 37°C for 90 minutes. Autoclave for 10 minutes at 110°C, then freeze at -80°C.

**Figure 2. Agglomeration of Geltrex^TM^ in PDO-ink due to delayed mixing. (a)** Agglomeration can be observed with naked eye. **(b)** Agglomeration according to mixing time. Mixing over 15 seconds can make Geltrex^TM^ agglomerate in the syringe, finally failing to be extruded. Yellow arrows indicate agglomeration in PDO-ink.

1. Cell Preparation: Centrifuge dissociated CRC cells (7.5 × 10⁶ cells) at 125 × g for 5 minutes; aspirate supernatant, leaving cell pellet.
2. Mixing PDO-Ink: Mix the cell pellet sequentially with 100 μL DPBS, 75 μL gelatin/HA mixture, and 75 μL Geltrex™. Gently pipette without creating bubbles. (Important: Complete mixing Geltrex™ within 15 sec. Delayed mixing can lead to agglomeration of Geltrex™, Figure 2)
3. Final Cooling: Load PDO-ink into sterile syringes, cover to maintain sterility. Place in an ice bath for at least 10 minutes before printing.

**4. Printing of Eba-PDOs**

1. Setup: Prepare bioprinting system. Cool printing chamber to 19°C, maintain humidity >70%. Load syringes, replacing needles with specific nozzles:

- PCL: Use nozzle SHN-0.2N, sterilize with 70% ethanol before use.
- PDO-Ink: Use nozzle SPN-0.12-0.65L, also sterilized with 70% ethanol.
- Bath-Ink: Use nozzle SHN-0.3N, sterilized prior to use.

1. Printing (Figure 3):

- PCL Wall: Print a 5 × 5 × 0.6 mm³ wall at 150 kPa using a 200 μm nozzle.
- Bath Ink Filling: Dispense bath-ink to fill PCL mold, ensuring uniform coverage.
- PDO-Ink Extrusion: Extrude at 0.01 μL/s for 1.5 s per 3D dot; print a 7×7 grid (49 dots total).

1. Crosslinking: Move printed constructs to ice for temporary crosslinking. Add 40 mM CaCl₂ solution dropwise, let sit for 30 minutes. Transfer to 24-well plate, continue culture.

**5. End-point Imaging**

- On day 14, take bright-field images of Eba-PDO constructs using a microscope for structural analysis.

**Tips for Reproducibility and Troubleshooting**

- Ensure all equipment is properly calibrated and maintained.
- Regularly monitor the temperature and humidity of solutions to ensure optimal conditions for successful bioprinting (Figure 4).
- Strictly maintain sterile conditions to prevent contamination throughout the process.
- Verify nozzle condition before each printing session to ensure accurate deposition. Particularly, bent nozzles can prevent steady extrusion of PDO-ink.
- Clean the nozzle for each printing batch.


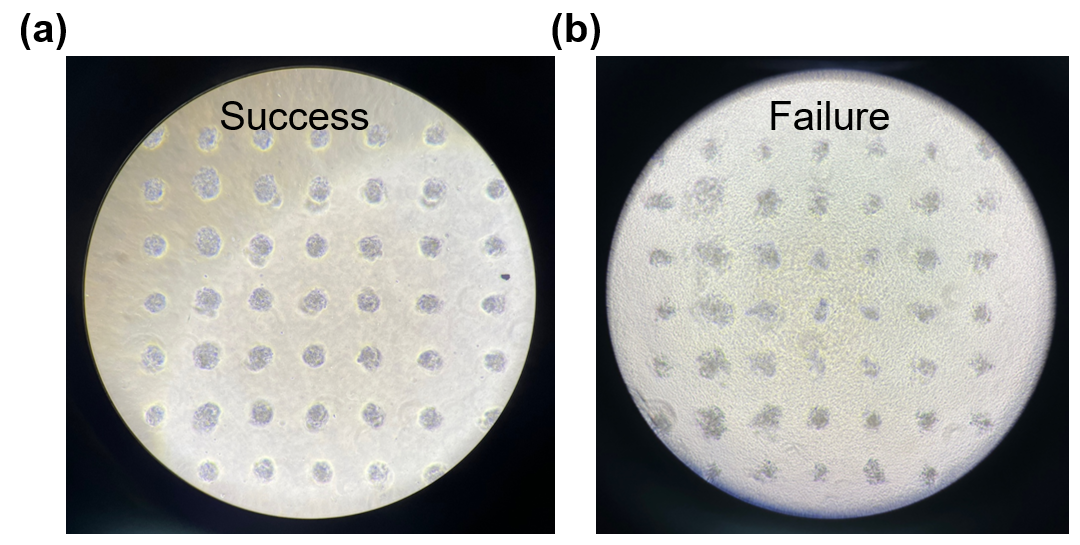

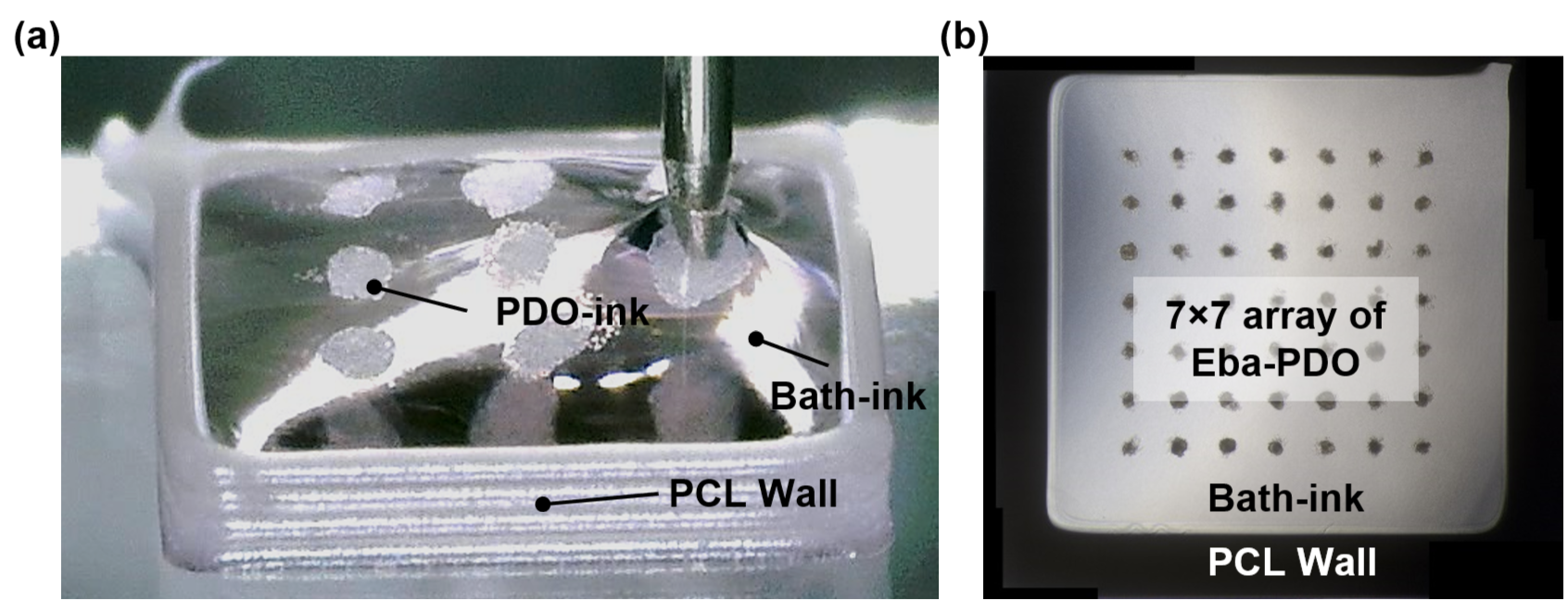


**Figure 4. Printing results of PDO array. (a)** Successful and **(b)** failed printing of Eba-PDO. Unstable temperature conditioning or agglomeration can induce failure of printing.

**Figure 3. Bioprinting process and a bioprinted construct including PCL wall, Bath-ink and PDO-ink. (a)** PDO-ink is being extruded in the alginate bath. **(b)** a bioprinted construct with 7×7 array of Eba-PDOs.
